# A Discrete Trialane with a Near-Linear Al_3_ Axis

**DOI:** 10.1021/jacs.4c10967

**Published:** 2024-11-26

**Authors:** Debabrata Dhara, Lukas Endres, Aritra Roy, Rian D. Dewhurst, Rüdiger Bertermann, Felipe Fantuzzi, Holger Braunschweig

**Affiliations:** † Institute for Inorganic Chemistry, Julius-Maximilians-Universität Würzburg, Am Hubland, 97074 Würzburg. Germany; ‡ Institute for Sustainable Chemistry & Catalysis with Boron, Julius-Maximilians-Universität Würzburg, Am Hubland, 97074 Würzburg, Germany; § Institute for Physical and Theoretical Chemistry, Julius-Maximilians-Universität Würzburg, Emil-Fischer-Str. 42, 97074 Würzburg, Germany; ∥ Department of Chemical and Energy Engineering, London South Bank University, 103 Borough Road, London SE1 0AA, U.K.; ⊥ School of Chemistry and Forensic Science, University of Kent, Park Wood Rd, Canterbury CT2 7NH, U.K.

## Abstract

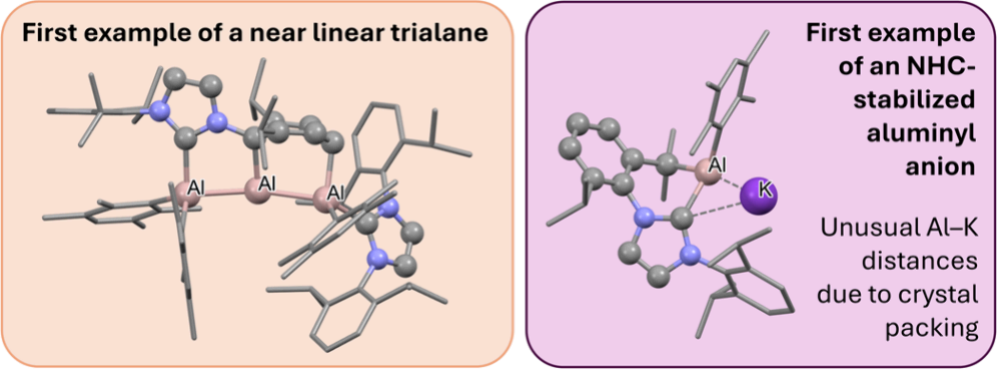

The presence of inherent electronic unsaturation in aluminum predominantly results in the formation of aluminum clusters, with very few examples of compounds containing discrete chains of aluminum atoms in existence. In this work, we present the successful synthesis and structural authentication of a highly unusual trialane species with a near-linear chain of three Al atoms, alongside a carbene-stabilized aluminyl anion ([LAlR_2_]^−^), an alternative product produced by varying the reaction conditions. Quantum-chemical calculations have been applied to elucidate the electronic structure and bonding of these novel compounds. Additionally, we successfully trapped a reaction intermediate using an alkyne, suggesting the intermediacy of a base-stabilized monomeric alumylene (LRAl:), which is also investigated through computational methods.

## Introduction

**Figure 1:**
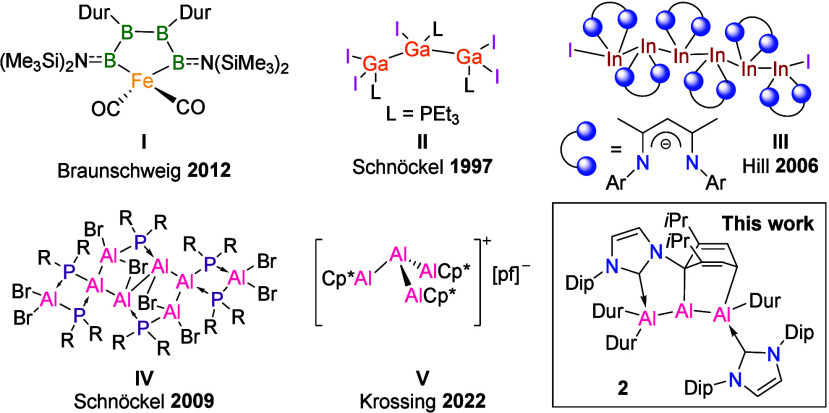
Selected examples of Group 13 homocatenated compounds. Definitions: R = *t*Bu; Cp* = C_5_Me_5_; pf = Al{C(CF_3_)_3_}_4_; Dur = 2,3,5,6-C_6_HMe_4_; Dip = 2,6-*i*Pr_2_C_6_H_3_.

The tendency for Group 13 elements to favor aggregated structures and clusters over homocatenated linear compounds is well-documented and is rooted in their inherent electron decifiency.^1,2^ Nonetheless, research into Group 13 dielement species (e.g., diboranes/-enes/-ynes in the case of boron) continues, thanks to their interesting properties and reactivity.^3−13^ Conversely, compounds containing discrete chains of Group 13 derivatives remain rare. The first linear triborane, B_3_[N(CH_3_)_2_]_5_ was synthesized by uncontrolled reduction of chloro(dimethyl amino)boranes.^13^ This method was later employed to synthesize B_6_(NMe_2_)_8_ and cyclo-B_6_(NR_2_)_6_ (R = Me, Et).^12−17^ However, the controlled formation of a homocatenated linear tetraborane, **I** (Figure 1), was first achieved by consecutive coupling of borylene units (RB:, R = duryl, N(SiMe_3_)_2_) stabilized by a transition metal center.^18^ Compounds containing chains of boron atoms are rare but this area has seen some recent progress, thanks to synthetic techniques such as the reductive coupling of haloboranes,^24^ borylene homocoupling^18^ and the hydroboration of diborenes.^19−23,25^ Catenates of the heavier Group 13 atoms are exceedingly rare, primarily owing to the challenges involved in their synthesis and stability. In 1997, Schnöckel and co-workers successfully generated a trimeric linear gallium complex, Ga_3_I_5_•3PEt_3_ (**II**, Figure 1), along with the digallane [(GaI_2_•PEt_3_)_2_], through the application of ultrasound to a mixture of gallium and diiodine in the presence of triethyl phosphine.^26^ In 2006, Hill and co-workers demonstrated the controlled formation of the six-atom indium homocatenate **III** (Figure 1) through a reaction involving a moderately congested β-diketiminate ligand with indium(I) iodide in the presence of the base K[N(SiMe_3_)]_2_.^27^ Following a similar strategy, Linti reported an amidinate-stabilized trigallane species in 2011.^28^

In the case of aluminum, the state of the art in homocatenation is less straightforward thanks to the atom’s high propensity for aggregation. In 1998, Schnöckel and co-workers isolated Cp*_3_Al_5_I_6_ (Cp* = [C_5_Me_5_]^−^), containing Al_2_ and Al_3_ units by the reaction of [Cp*Al]_4_ with AlI_3_ in absence of coordinating solvent or ligands.^29^ A further method for obtaining oligomeric aluminum species involved the self-condensation or co-condensation of subvalent aluminum in the presence of donor ligands.^30^ Similarly, co-condensation of AlBr (toluene:THF, 3:1) with a small excess of *t*BuLi led to the formation of an Al_8_ core where a central Al_6_Br_4_(PR_2_)_4_ motif is stabilized by two AlBr_2_PR_2_ units (**IV**, Figure 1).^31^ Very recently, Krossing et al. reported the Al_4_ cluster cation [Al(AlCp*)_3_]^+^[pf]^−^ ([pf]^−^ = [Al(OC(CF_3_)_3_)_4_]^−^; **V**, Figure 1), its core resembling a branched tetraalane, by combining [(AlCp*)_4_] with Li[pf].^32^ Quantum-chemical findings suggested that cyclic trinuclear aluminum compounds tend to be more stable than their linear counterparts,^33,34^ and this is borne out by work from Power and co-workers that showed that the reduction of ArAlI_2_ (Ar = 2,6-Mes_2_-C_6_H_3_, 2,6-Tip_2_-C_6_H_3_, Mes = 2,3,5-C_6_H_2_, Tip = 2,3,5-C_6_H_2_) readily yielded a dianionic cyclic cluster, Na_2_[(AlAr)_3_].^35,36^ Despite these landmark syntheses of low-valent aluminum species, neutral molecules containing discrete, noncluster Al_3_ units remain unknown.

In this study, we present the synthesis of a stable linear trialane, **2** (Figure 1), that results from the reduction of an *N*-heterocyclic carbene (NHC)-stabilized aluminum dibromide in hexane. This compound can be viewed as an aluminum analog of propylene, deactivated through a [2 + 4] cycloaddition with a peripheral aromatic group of the carbene donor. Additionally, the reduction of the same aluminum dibromide adduct with a 5-fold excess of reducing agent furnished a hitherto unknown NHC-stabilized aluminyl anion. It is highly probable that the formation of both compounds occurs initially through the creation of an NHC-stabilized alumylene species. This proposition is substantiated by trapping of this species *in situ* with bis(trimethylsilyl)acetylene. To gain a comprehensive understanding of the resulting compounds, theoretical studies were conducted to elucidate their bonding and electronic properties.

## Results and Discussion

**Scheme 1:**
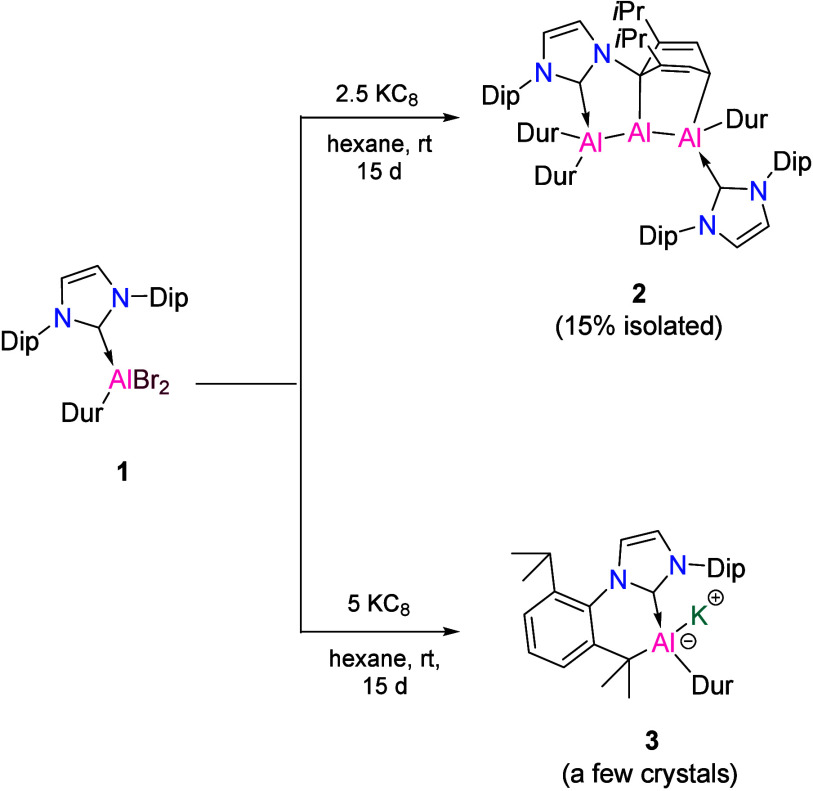
Synthesis of Compounds **2** and **3** from **1**

**Scheme 2:**
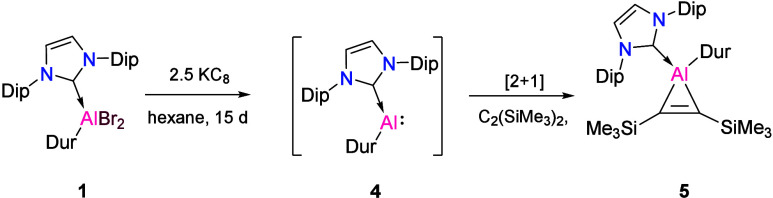
Synthesis of Compounds **4** and **5** from **1**

Trialane **2** was synthesized through a two-step process. In the initial step, [(Et_2_O)AlBr_2_Dur] (Dur = 2,3,5,6-C_6_HMe_4_)^37^ was treated with the NHC 1,3-bis(2,6-di*iso*propylphenyl)-imidazol-2-ylidene (IDip),^38^ in hexane at room temperature, resulting in the formation of IDip-stabilized durylaluminum dibromide (**1**; Scheme 1). In the subsequent step, reduction of compound **1** was carried out using 2.5 equiv of KC_8_ in hexane, yielding a red solution. After purification, red crystals of compound **2** were obtained in a 15% yield (Scheme 2). It is noteworthy that a similar reduction of carbene-coordinated aryldihaloalanes had previously produced a range of different products, including a dialumene,^39^ a self-stabilized dialuemene,^40^ diverse dialanes,^41^ and a diradical dialumene.^37^ Compound **1** was characterized using standard spectroscopic, analytical, and crystallographic techniques. In contrast, compound **2** exhibits high instability in most common solvents, except for hexane and pentane. Therefore, its characterization relied on solid-state NMR and single-crystal X-ray diffraction.

**Figure 2:**
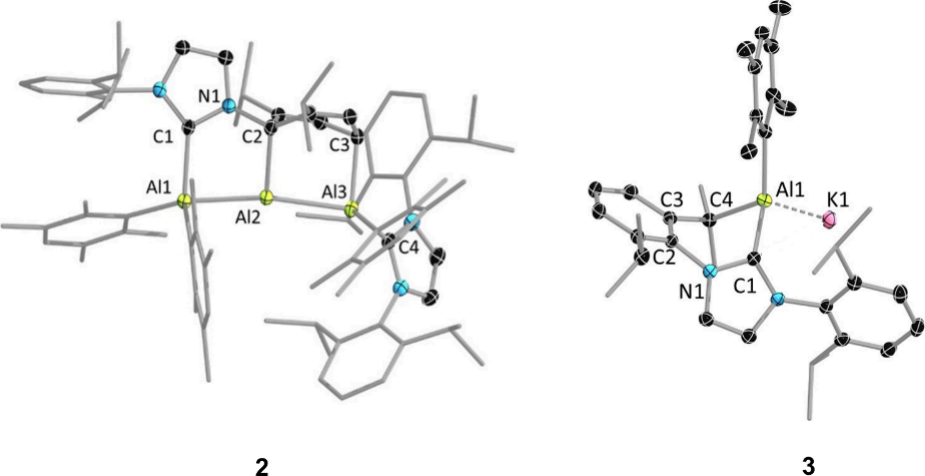
Solid-state molecular structures of **2** and **3**, with thermal ellipsoids at the 50% probability level. All hydrogen atoms are omitted for clarity. Selected bond lengths [Å] and bond angles [deg] for **2**: Al1–Al2 2.5931(5), Al2–Al3 2.5911(5), Al1–C1 2.1294(12), Al2–C2 2.0892(12), Al3–C3 2.0808(13), Al3–C4 2.1012(13); Al1–Al2–Al3 161.754(19). For **3**: Al1–K1 2.2064(7), Al1–C1 2.0631(17), Al1–C4 2.0313(17), Al1–C_Dur_ 2.0101(17); C4–Al1–C_Dur_ 115.19(7), C4–Al1–C1 89.27(7), C1–Al1–C_Dur_ 124.71(7), K1–Al1–C_Dur_ 110.90(5), K1–Al1–C1 101.07(4).

The solid-state structure of compound **2** shows the molecule to possess a near-linear Al_3_ core, the central Al atom of which is nearly T-shaped (Figure 2). The two terminal aluminum atoms within this core have clear oxidation states of +2, whereas the central aluminum atom assumes a + 1 oxidation state. This is further confirmed by computational calculations using the localized orbital bonding analysis (LOBA)^42^ as implemented in Multiwfn 3.8,^43^ which yield oxidation states of +2 for the terminal aluminum atoms and +1 for the central atom, resulting in a total oxidation state of +5 for the Al_3_ core. The Al–Al bonds in this compound are quite similar in length (Al1–Al2:2.5931(5) Å; Al2–Al3:2.5911(5) Å; Figure 2), falling within the range of typical Al–Al single bond distances (ranging from 2.50 to 2.95 Å).^44^ Notably, these bonds are significantly longer than those observed in the dialumene compound [(NHC)(Tip)Al = Al(Tip)(NHC)] (Al = Al: 2.4039(8) Å, Tip = 2,4,6-C_6_H_2_*i*Pr_3_, NHC = 1,3-di*iso*propyl-4,5-dimethylimidazol-2-ylidene).^39^ Furthermore, the three aluminum atoms in compound **2** exhibit a slight deviation from linearity (Al1–Al2–Al3 = 161.754(19)°, calcd. 163.4°), with the bonding angles of the central Al2 atom deviating significantly from the typical sp^2^ angle of 120° (Al1–Al2–C2 = 90.04(4)°, calcd. 90.4°; Al3–Al2–C2 = 94.03(4)°, calcd. 93.6°). The Al–C bond distances are nearly identical, with Al2–C2 measuring 2.0892(12) Å and Al3–C3 at 2.0808(13) Å. These bond lengths are slightly shorter than those of Al1–C1 (2.1294(12) Å) and Al3–C4 (2.1012(13) Å), both of which are formally dative bonds involving the NHC donors. In the solid-state ^13^C{^1^H} NMR spectrum of **2**, two broad resonances were observed at 179.4 and 217.0 ppm and were assigned to the carbene carbon nuclei. Signals corresponding to the Al-bound carbon nuclei of the dearomatized arene ring could not be identified, presumably due to quadrupolar broadening.

**Figure 3:**
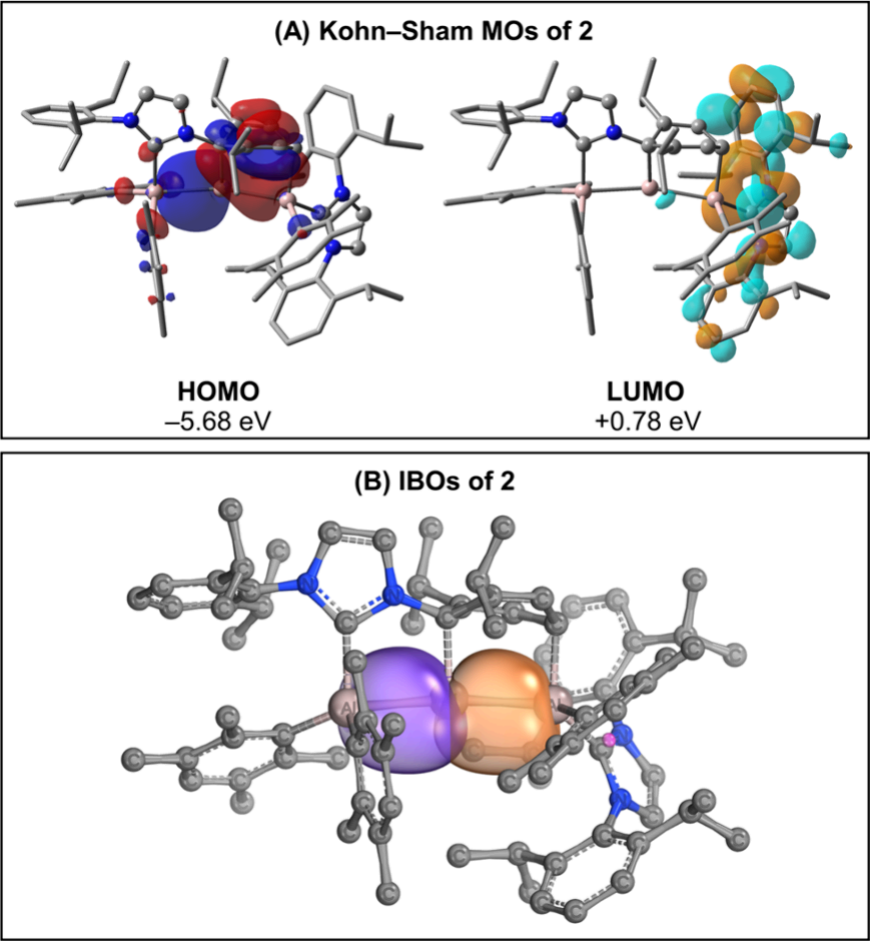
(A) Canonical Kohn–Sham molecular orbitals of compound **2**. Calculations were performed at the ωB97X-D/Def2-TZVP level of theory. Hydrogen atoms are omitted for clarity. The HOMO–LUMO gap at this level of theory is 6.46 eV. (B) Intrinsic bond orbitals of compound **2** depicting the Al–Al bonds.

To gain further insights into the electronic structure of **2**, we performed density functional theory (DFT) calculations at the ωB97X-D/Def2-TZVP level, using structures optimized at ωB97X-D/Def2-SVP. Initially, we conducted calculations for both singlet and triplet states, yielding vertical and adiabatic singlet–triplet gaps of 53.2 kcal mol^–1^ and 37.7 kcal mol^–1^, respectively. These results confirm that the system is indeed a closed-shell singlet, with no biradicaloid character, contrasting with the Al–Al bond systems recently obtained and characterized by our group.^37,45^ Subsequently, we analyzed the Mayer bond orders (MBOs) of the system. The Al–Al bonds both exhibit an MBO value of 0.95, indicating the absence of π interactions across these bonds. This is corroborated by the examination of the canonical Kohn–Sham molecular orbitals (MOs) and intrinsic bond orbital (IBO)^46^ analysis (Figure 3). The HOMO of **2** consists primarily of σ contributions from the Al–Al bonds, along with additional contributions from the phenyl ring attached to the Al core, while the LUMO is located on the IDip ligands. The IBOs associated with the Al–Al bond further confirm the absence of π components. These findings provide a comprehensive understanding of the bonding nature in **2**, emphasizing the dominance of σ interactions and the lack of π character in the Al–Al bonds.

In a variation of the above reaction, subjecting **1** to an excess of the reducing agent KC_8_ (five equivalents) resulted in the formation of a few crystals of aluminyl anion **3** (Scheme 1). It is worth noting that isolable aluminyl anions are very rare species. Aldridge’s pioneering work led to the isolation of the first aluminyl anion, sparking subsequent advances in this field.^47^ Most known examples are stabilized within a robust chelating, dianionic ligand environment and are cyclic in nature.^48^ Liptrot and co-workers have recently reported the first observation of an aluminyl anion based on nonchelating ligands, obtained by a potassium-mediated reduction of [AlI{N(Dip)SiMe_3_}_2_] (Dip = 2,6-*i*Pr_2_C_6_H_3_).^49^ Nevertheless, to date, there are no reports of tricoordinate carbene-stabilized aluminyl anions of the form [LAlR_2_]^−^. This absence is likely attributable to the inherent proclivity of this anion to undergo disproportionation, which is perhaps also responsible for the notably low yield of **3**. Compound **3** could only be characterized by solid-state molecular structure determination (Figure 2). During its formation, one of the isopropyl C–H bonds of the IDip donor is activated, furnishing a six-membered ring. The Al1–C3 (2.0313(17) Å) and Al1–C_Dur_ (2.0101(17)Å) bonds are similar in length and are comparable to typical Al–C bond distances but slightly shorter than the Al–C_NHC_ bond (Al1–C1 2.0631(17) Å). An intriguing observation was the aluminum-to-potassium bond distance (Al1–K1 2.2064(7) Å), which is much shorter than a comparable reported alkyl-substituted aluminum anion (3.4549(5) Å)^50^ and other reported aluminum anions (3.5346(8)–3.7053(9) Å).^47,51−54^ Additionally, this bond distance is notably shorter than the sum of the covalent radii of aluminum and potassium (3.28 Å).^50^

**Figure 4:**
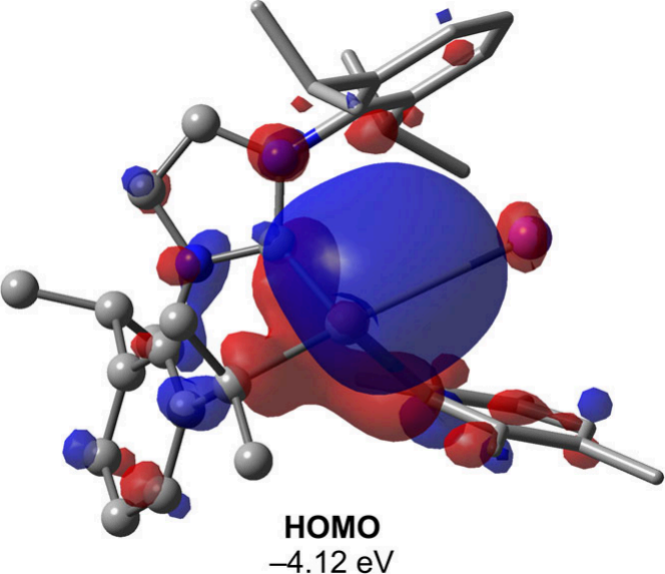
HOMO of compound **3**. Calculations were performed at the ωB97X-D/Def2-TZVP level of theory. Hydrogen atoms are omitted for clarity. The HOMO–LUMO gap at this level of theory is 4.62 eV.

Given the unique nature of compound **3**, we conducted DFT calculations to gain insights into its electronic structure and compared it with Yamashita’s alkyl-substituted aluminum anion.^49^ Surprisingly, while the optimized Al–K bond length in Yamashita’s system was 3.378 Å—slightly shorter than the experimental crystal structure—the Al–K distance in compound **3** increased to 3.277 Å, with the K atom centrally located between the two Ph groups. The MBO analysis revealed an Al–K bond order of 0.31 in compound **3**, nearly identical to that of Yamashita’s system (0.30). Additionally, compound **3** exhibited a significantly smaller HOMO–LUMO gap (4.62 eV) compared to compound **2** (6.46 eV) and Yamashita’s system (5.76 eV). A similar trend was observed in the vertical and adiabatic singlet–triplet gaps, which were 40.8 kcal mol^–1^ and 35.5 kcal mol^–1^ for Yamashita’s system, respectively, and 26.3 kcal mol^–1^ and 10.8 kcal mol^–1^ for compound **3**. The HOMO of **3**, depicted in Figure 4, underscores the “aluminum anion” nature of the system. Taken together, these results suggest that the unusually short Al–K contact observed in the crystals of compound **3** is likely due to a crystal packing effect.

**Figure 5:**
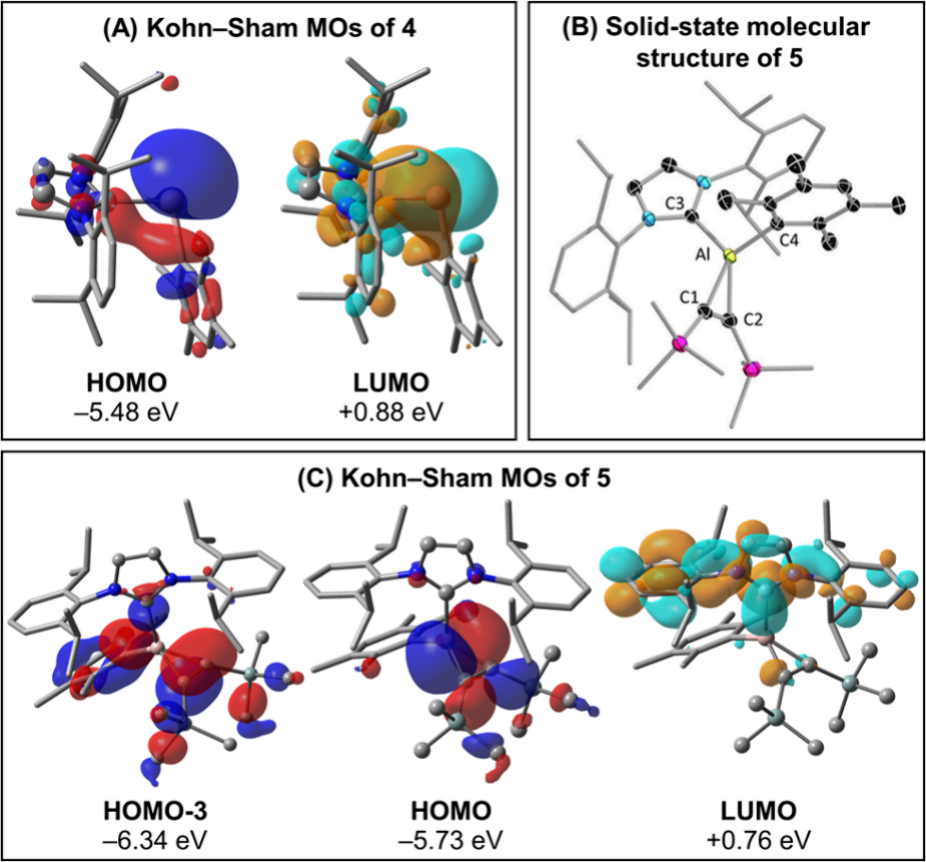
(A) Canonical Kohn–Sham molecular orbitals of **4**. (B) Solid-state molecular structure of **5**. with thermal ellipsoids at the 50% probability level. All hydrogen atoms are omitted for clarity. Selected bond lengths [Å] and bond angles [deg]: Al–C1 1.936(2), Al–C2 1.948(2), C1–C2 1.365(3), Al–C3 2.0492(2), Al–C4 1.997(2); C1–Al–C2 41.16(9). (C) Canonical Kohn–Sham molecular orbitals of **4**. Calculations were performed at the ωB97X-D/Def2-TZVP level of theory. Hydrogen atoms are omitted for clarity. The HOMO–LUMO gaps at this level of theory are 6.36 eV for **4** and 6.49 eV for **5**.

The formation of the trialane compound **2** and the aluminyl anion **3** from compound **1** suggests that an alumylene intermediate is formed during the course of the reaction. Indeed, the reversible dissociation of dialumenes (RAl = AlR) into alumylenes (RAl:) is also a well-established phenomenon in aluminum chemistry.^27,40,55,56^ In 2021, Krämer and Cowley reported the reversible dissociation of an amidophosphine-supported dialumene into the corresponding alumylene.^55^ This alumylene was effectively trapped with an alkyne, leading to the formation of an intriguing aluminocyclopropene. In recent work, we have also used this strategy to capture a transient alumylene species.^57^ In an attempt to trap the base-stabilized monomeric alumylene **4**, we conducted the reduction of compound **1** in the presence of bis(trimethylsilyl)acetylene, resulting in the generation of aluminocyclopropene **5** (Scheme 2). This provides strong evidence in favor of the proposed formation of alumylene **4** in the course of the reaction to form the trialene compound **2**. The molecular structure of **5** (Figure 5) showed a distinct C=C bond with a C1–C2 bond length of 1.365(2) Å, similar to other aluminocyclopropene rings.^55,57^

We became interested in further investigating the electronic structure of **4** through DFT calculations, specifically examining its differences when a different carbene ligand, such as a cyclic(alkyl)(amino) carbene (CAAC), replaces the NHC IDip in **4**, resulting in **4**^**CAAC**^. This comparison is particularly relevant in the context of their boron-based counterparts, borylenes, which we have recently studied extensively from a computational perspective, focusing on CAAC- and NHC-stabilized variants.^58^

CAAC is a better π-acceptor than IDip, making it more suitable for stabilizing radicals and biradicals.^59,60^ Surprisingly, however, carbene-stabilized borylenes of type RLB: with CAAC ligands exhibit a singlet ground state, whereas their NHC counterparts are all triplet ground states.^61^ This explains the discovery of various diborenes featuring IDip and other NHCs, which can be considered dimerization products of triplet borylenes. For aluminum, the situation is different. Both **4** and **4**^**CAAC**^ have singlet ground states and feature bent structures. The Al lone pair of **4** appears as the HOMO of the system, as shown in Figure 5A. In contrast, the LUMO of **4** is mostly composed of a twisted Al–C^NHC^ π orbital. Additionally, the vertical and adiabatic singlet–triplet gaps of **4** are 31.9 kcal mol^–1^ and 17.2 kcal mol^–1^, respectively, which are larger than those of **4**^**CAAC**^, with values of 22.9 kcal mol^–1^ and 4.8 kcal mol^–1^.

The relatively low adiabatic singlet–triplet gap of **4** (c.f. the value of 37.7 kcal mol^–1^ for **2**, for example) can explain not only its enhanced reactivity but also its trapping with bis(trimethylsilyl)acetylene, leading to compound **5**, whose solid-state structure is shown in Figure 5B. The stability of **5** is comparable to that of **2**, with vertical and adiabatic singlet–triplet gaps of 52.4 kcal mol^–1^ and 33.1 kcal mol^–1^, as well as a HOMO–LUMO gap of 6.49 eV, the largest among the compounds investigated herein. The canonical Kohn–Sham MOs of **5**, illustrated in Figure 5C, indicate that the HOMO is primarily localized on the C_2_Al ring. According to the charge decomposition analysis^62^ method as implemented in Multiwfn 3.8,^43^ the HOMO predominantly consists of the in-plane antibonding C–C π* orbital of the acetylene moiety (62%). The second most important contribution (20%) originates from the LUMO of the alumylene fragment **4**. In turn, the LUMO of **5** is situated in the π-space of the IDip ligand and extends to the Dip groups. Finally, HOMO–3 depicts the C–C π bond of the aluminocyclopropene ring. Altogether, these results highlight the intricate electronic structure of compounds **4** and **5**, emphasizing the unique stabilization provided by the C_2_Al ring and the IDip ligand, and suggesting new avenues for exploring the reactivity and design of aluminum-based π-complexes.

To examine whether compound **5** could theoretically act as a source for the base-stabilized monomeric alumylene **4**, we performed additional computations to examine the interaction energy between **4** and bis(trimethylsilyl)acetylene. At the ωB97X-D/Def2-TZVP level of theory, the Gibbs free energy for the dissociation of **5** into **4** plus the alkyne is calculated to be +27.0 kcal/mol, suggesting that the formation of **4** from **5** could potentially be achievable with thermal input. However, experimental evidence shows that compound **5** is unstable in benzene, decomposing even at room temperature. Due to this instability, further heating would likely lead to complete decomposition rather than the clean formation of **4**.

**Scheme 3:**
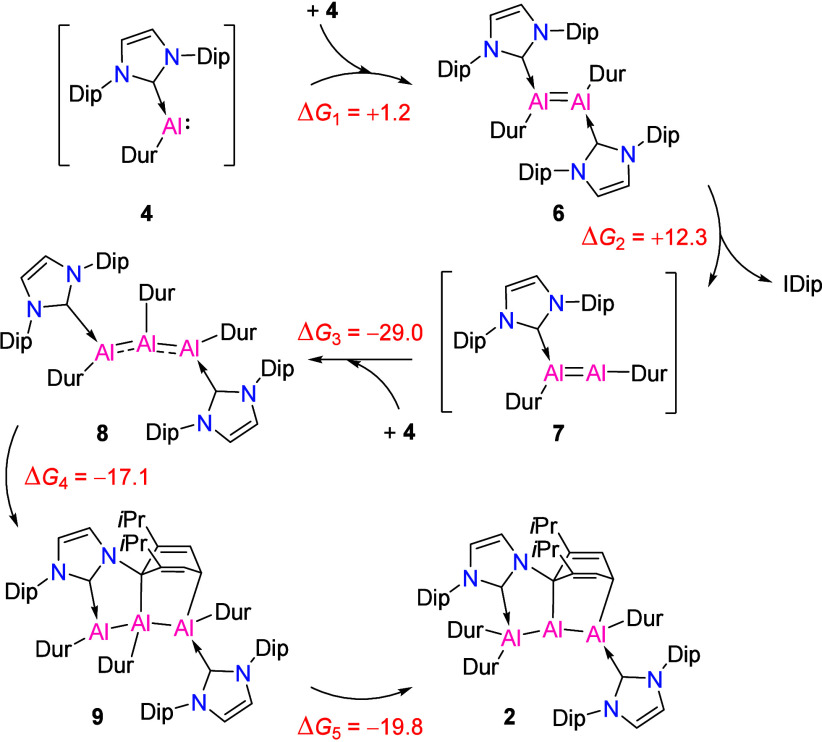
Proposed Mechanism for the Formation of **2** from **4** The computed free energies of reaction (kcal/mol) for each step are also shown.

Finally, in order to better understand the mechanism of the formation of **2** from **4**, we conducted a thorough computational investigation focused on identifying plausible intermediates and energetically favorable pathways. Given the large size of the systems involved, a complete mechanistic investigation of all possible transition states was impractical and beyond the scope of this study. Instead, we focused our efforts on key intermediates and energetically viable reaction steps to propose a reasonable mechanistic pathway. The proposed mechanism is illustrated in Scheme 3.

Our computational analysis began by examining the dimerization of **4** to form the dialumene species **6**. This reaction was found to be slightly endergonic, with a free energy difference of Δ*G*_1_ = +1.2 kcal/mol in hexane solution. From this intermediate, we propose that one of the carbene ligands detaches, forming intermediate **7**. The free energy change for this process is Δ*G*_2_ = +12.3 kcal/mol. The formation of the free IDip, which is supported by our NMR observations, aligns well with the computational results, confirming that this intermediate likely plays a key role in the reaction mechanism. Intermediate **7** is characterized by a dicoordinate aluminum center, which opens up the possibility for further reactivity.

The subsequent reaction of **7** with a third molecule of **4** leads to the formation of the trialane compound **8** (Δ*G*_3_ = −29.0 kcal/mol). Notably, this reaction is more exergonic than the alternative reaction of **7** with the free IDip, indicating that the pathway involving **7** → **8** is more favorable. Compound **8**, which features only tricoordinate aluminum atoms, presents two plausible routes to the formation of **2**. In the first pathway, one of the Dip substituents undergoes activation, resulting in a cycloaddition reaction that forms intermediate **9** (Δ*G*_4_ = −17.1 kcal/mol). The final step in this pathway involves the transfer of the central Dur substituent to the terminal aluminum, completing the formation of **2** with a free energy change of Δ*G*_5_ = −19.8 kcal/mol. The total free energy for the reaction, where three molecules of **4** yield compound **2** and a free IDip ligand, is Δ*G*_reac_ = −52.5 kcal/mol.

An alternative pathway was also considered, in which the Dur migration occurs before the cycloaddition. This pathway leads to intermediate **9’** (see Figure S12 in the SI). However, the formation of **9’** is energetically unfavorable (Δ*G*_4_^’^ = +26.0 kcal/mol), making this intermediate less stable than **9** by +43.1 kcal/mol. Therefore, based on these calculations, we propose that the cycloaddition occurs prior to Dur migration, as this pathway is energetically more favorable and involves more suitable intermediates.

## Conclusions

In summary, we report the synthesis and isolation of the first example of a neutral nonhypercoordinate trialane chain compound, as well as a new aluminyl anion. Quantum chemical calculations have demonstrated that both systems possess closed-shell singlet multiplicities, confirming the absence of any biradicaloid character. Additionally, we successfully trapped a plausible reaction intermediate in the trialane formation through the reduction of IDip-stabilized durylaluminum dibromide in the presence of an alkyne. This trapping product suggests the formation of a base-stabilized monomeric alumylene (LRAl:) in the course of the reaction, whose electronic structure and reactivity were further investigated through computational calculations. We hope that this work will pave the way to the synthesis of other compounds bearing discrete chains of aluminum atoms, a conceptually simple but practically very challenging class of compounds.

## Supporting Information Available

Experimental section, plots of NMR and UV–vis spectra for new compounds, and complete details of crystallographic experiments and computational calculations (PDF)
Compiled coordinates of all calculated structures (XYZ)

## Supplementary Material

ja4c10967_si_001.pdf

ja4c10967_si_002.xyz
