# Supplementary material for: A Discrete Trialane with a Near-Linear Al3 Axis
Source: J Am Chem Soc. 2024 Nov 26;146(49):33536–42. doi: 10.1021/jacs.4c10967 (PMC11638959; doi:10.1021/jacs.4c10967)
Supplement: Supplementary file 1 — ja4c10967_si_001.pdf [file ja4c10967_si_001.pdf]

Supporting information for:

A Discrete Trialane with a Near-Linear Al_3 Axis

Debabrata Dhara,[1,2] Lukas Endres,[1,2,3] Aritra Roy,[4] Rian D. Dewhurst,[1,2] Rüdiger Bertermann,[1,2] Felipe Fantuzzi,[5*] Holger Braunschweig[1,2*]

[1] Institute for Inorganic Chemistry, Julius-Maximilians-Universität Würzburg, Am Hubland, 97074 Würzburg, Germany.

[2] Institute for Sustainable Chemistry & Catalysis with Boron, Julius-Maximilians-Universität Würzburg, Am Hub-land, 97074 Würzburg, Germany.

[3] Institute for Physical and Theoretical Chemistry, Julius-Maximilians-Universität Würzburg, Emil-Fischer-Str. 42, 97074 Würzburg, Germany.

[4] Department of Chemical and Energy Engineering, London South Bank University, 103 Borough Road, London SE1 0AA, UK

[5] School of Chemistry and Forensic Science, University of Kent, Park Wood Rd, Canterbury CT2 7NH, UK.

*Correspondence author. Email: h.braunschweig@uni-wuerzburg.de; f.fantuzzi@kent.ac.uk

General experimental considerations

All syntheses were carried out under an atmosphere of argon or nitrogen in argon-filled gloveboxes or using standard Schlenk techniques. IDip (IDip =1,3-bis(2,6-diisopropylphenyl)-imidazol-2-ylidene),[1] and DurLi (Dur = 2,3,5,6-C_6HMe_4),[2] [$(Et_2O)AlBr_2(Dur)$][3] were prepared according to literature procedures. Deuterated solvents were dried over molecular sieves and degassed by three freeze-pump-thaw cycles before use. All other solvents were distilled and degassed from appropriate drying agents. Both deuterated and non-deuterated solvents were stored under argon over activated 4 Å molecular sieves. All NMR spectra were obtained from a Bruker Avance III HD 300 NMR spectrometer ($^{13}C\{^1H\}$: 75.5 MHz) or a Bruker Avance I 400 NMR spectrometer (^1H: 400.6 MHz, $^{13}C\{^1H\}$: 100.6 MHz) at 298 K, unless otherwise stated. Chemical shifts (δ) are reported in ppm and internally referenced to the carbon nuclei ($^{13}C\{^1H\}$) or residual protons (^1H) of the solvent. Resonances are given as singlet (s), doublet (d), triplet (t), quartet (q), septet (sept) or multiplet (m). The solid-state $^{13}C\{^1H\}$ CP/MAS, $^{13}C\{^1H\}$ CPPI/MASNMR spectra were recorded at 22 °C with a Bruker Avance NEO 400 NMR spectrometer with bottom layer rotors of ZrO_2 (outer diameter 2.5 mm, Vespel bottom cap and Vespel rotor cap) containing approximately 11 mL of sample spinning the rotor at different speeds between 13.5 and 15 kHz (^1H, 400.13 MHz; ^{13}C, 100.61 MHz; ^{27}Al, 104.26131 MHz; DP = direct polarization, CP = cross-polarization, PI = polarization inversion, HPdec = ^1H high-power decoupling, MAS = magic-angle spinning). The polarization inversion time in the $^{13}C\{^1H\}$ CPPI/MAS solid-state NMR spectra was 70 ms. All chemical shifts were calibrated by setting the ^{13}C low-field signal of adamantane to δ = 38.48 ppm by adjusting the field value of the spectrometer according to the IUPAC recommendations with $\Xi[^{13}C]$ = 25.145020 MHz and $\Xi[^{27}Al]$ = 26.056859.[4] High-resolution mass spectrometry (HRMS) data were obtained from a Thermo Scientific Exactive Plus spectrometer using a LIFDI 700 source from Linden CMS. UV-vis spectra were measured on a METTLER TOLEDO UV-vis Excellence UV5 spectrophotometer at room temperature. Infrared spectra were measured on a Bruker Optics Alpha FT-IR spectrometer in the ATR mode (w denotes weak, m denotes medium, s denotes strong, vw denotes very weak and vs denotes very strong absorption bands). Unless otherwise noted, a baseline correction was applied to IR spectra.

Synthesis of [(IDip)AlBr$_2$(Dur)] (1)

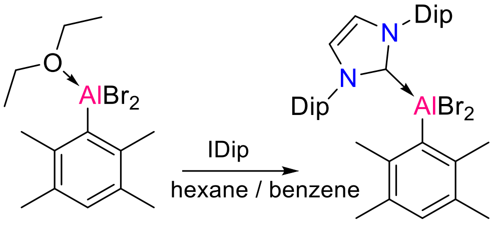

Hexane and benzene (1:1, total 15 mL) were rapidly added to a mixture of [(Et$_2$O)AlBr$_2$(Dur)] (1.50 g, 3.80 mmol) and IDip (1.48 g, 3.80 mmol) at room temperature under vigorous stirring. After 30 minutes of stirring, a white precipitate was subsequently separated by centrifugation and filtration from the resulting colorless solution. It was then washed twice with pentane to afford **1** as a white powder (2.60 g, 94% yield). X-ray quality single crystals were grown from a saturated benzene solution. ^{1}H NMR (400 MHz, C_6D_6): δ = 0.93 (d, ^3J = 7 Hz, 12H, CH(C*H*$_3$)$_2$, from Dip), 1.26 (d, ^3J = 7 Hz, 12H, CH(C*H*$_3$)$_2$, from Dip), 2.06 (s, 6H, C*H*$_3$, Dur), 2.26 (s, 6H, C*H*$_3$, Dur), 2.89 (sept, ^3J = 7 Hz, 4H, C*H*(CH$_3$)$_2$, Dip), 6.43 (s, 2H, C*H*=C*H*, IDip), 6.80 (s, 1H, Ar-*H*, Dur), 7.03 (s, 2H, Ar-*H*, Dip), 7.05 (s, Ar-*H,* Dip), 7.20 (t, ^3J = 7 Hz, 2H, Ar-*H,* Dip) ppm. ^{13}C{^{1}H} NMR (101 MHz, C_6D_6) δ = 21.1 (*C*H$_3$, Dur), 22.9 (*C*H$_3$, Dip), 25.7 (*C*H$_3$, Dur,), 26.9 (*C*H$_3$, Dip), 29.7 (C*H*(CH$_3$)$_2$, Dip), 124.9 (Ar-*C*H, Dip), 126.2 (*C*H=*C*H, IDip), 129.1 (Ar-*C*$_q$), 131.9 (*C*H, *p*-Ar-*C*H, Dip), 132.9 (Ar-*C*H, Dur), 133.1 (Ar-*C*$_q$), 135.3 (Ar-*C*$_q$), 143.5 (Ar-*C*$_q$), 146.7 (Ar-*C*$_q$), 167.0 (carbene carbon, observed in HMBC) ppm. Resonances for the Al-bound carbon nuclei and ^{27}Al were not observed due to quadrupolar broadening by the ^{27}Al nucleus. IR (ATR, 20 °C) ṽ cm^{-1}: 3162 (w), 3133 (w), 3100 (w), 1568 (w), 1461 (vs), 1443 (vs), 1404 (s), 1387 (ms), 1370 (ms), 1330 (m), 1058 (w), 996 (w), 933 (m), 877 (m), 809 (vs), 770 (vs), 718 (w), 673 (w), 633 (w). Elemental analysis (%) calcd for $C_{37}H_{49}AlBr_2N_2$ (708.6020): C, 62.72; H, 6.97; N, 3.95. Found: C, 62.64; H, 7.07; N, 3.84.

Synthesis of 2

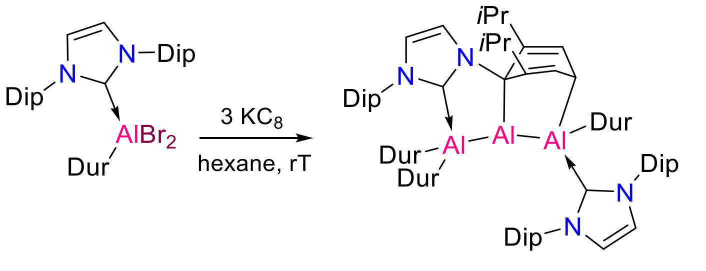

1 **2** (15% isolated)

Inside a glove box, **1** (0.500 g, 0.705 mmol) and freshly prepared potassium graphite (0.287 g, 2.11 mmol) were added to a vial and the mixture was cooled to –35 ºC followed by the addition of 15 mL of hexane.

The mixture was then allowed to stir for 15 days at room temperature. A ^{1}H NMR spectrum of the crude reaction mixture showed full consumption of **1** and peaks for free IDip. The solution was filtered and the solid was washed with hexane twice (10 mL each). The combined hexane extract was evaporated to yield a red solid, which was then extracted with 15 mL of hexane. The resulting hexane solution was concentrated to around 7 mL and stored in the glovebox freezer (–35 ºC) to obtain dark red crystals of **2**. Yield: 45.0 mg (15%). ^{13}C NMR (CP/MAS, 15.0 kHz): δ = 19.5 (CH_3), 21.0 (CH_3), 23.4 (CH_3), 24.0 (CH_3), 25.0 (CH_3), 28.1 (CH_3), 28.9 (CH_3), 29.8 (*C*H), 30.4 (*C*H), 84.9 (H*C*=*C*H, NHC), 110.1 (H*C*=*C*H, NHC), 120.4 (Ar-*C*H), 122.9 (Ar-*C*H), 123.4 (Ar-*C*H), 124.9 (Ar-C_q) 125.5 (Ar-*C*H), 128.1 (Ar-*C*H), 129.6 (Ar-C_q), 134.5 (Ar-C_q), 135.6 (Ar-C_q), 137.5 (Ar-C_q), 139.4 (Ar-C_q), 142.7 (Ar-C_q), 144.1 (Ar-C_q), 146.2 (Ar-C_q), 147.5 (Ar-C_q), 179.4 (C_q, NHC), 217.0 (C_q, NHC) ppm. UV-vis (hexane, 298 K) λ_{max} = 462 nm. IR (ATR, 20 °C) $\tilde{\nu}$ cm^{-1}: 1597 (w), 1557 (w), 1461 (vs), 1409 (s), 1387 (s), 1360 (m), 1324 (w), 1262 (w), 1198 (m), 1182 (m), 1115 (m), 1103 (m), 1063 (m), 1012 (m), 928 (m), 905 (s), 865 (w), 843 (w), 809 (s), 752 (vs), 718 (w), 684 (w). Elemental analysis (%) calcd for $C_{84}H_{111}Al_3N_4$ (1256.82): C, 80.21; H, 8.90; N, 4.45. Found: C, 80.58; H, 9.01; N, 4.43.

*Comments: Crystalline **2** is partially soluble in benzene, and toluene. Upon complete dissolution in benzene and toluene, **2** undergoes decomposition, resulting in a colorless solution with an unidentified mixture of products. In THF, $CHCl_3$, CH_2Cl_2, and CH_3CN, **2** decomposes almost immediately. Occasionally, white crystals of IDip also accompany crystals of **2**, which can be removed by washing with hexane. At ambient temperature, **2** slowly decomposes into a colorless solid. The red crystals also become colorless powder upon applying a vacuum in a Schlenk line for an hour. The crystals of compound **2** are slightly soluble in hexane, which is sufficient for obtaining UV-vis spectra. We attempted to record NMR in methylcyclohexane-d_{14}, but crystalline **2** is not very soluble in methylcyclohexane (Figure S1A). After shaking for an hour, we observed a red color (Figure 1B). However, in the NMR spectra, we found only free carbene, indicating decomposition of compound **2**. Overnight, compound **2** completely decomposed (the red color turned completely colorless, Figure 1C). We also observed a similar phenomenon in cyclohexane-d_{12}. This phenomenon also explains the low yield of **2**.*

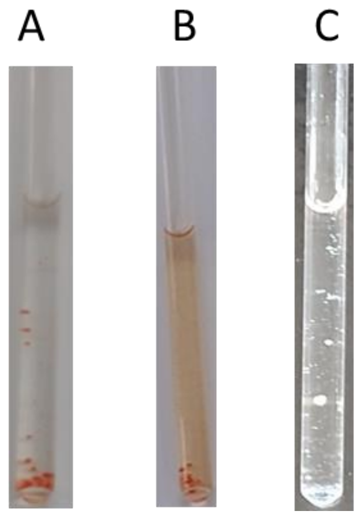

Figure S1. Picture of **2** in methylcyclohexane-d_{14} immediately after mixing (A), after one hour (B) and after overnight (C).

Synthesis of 3

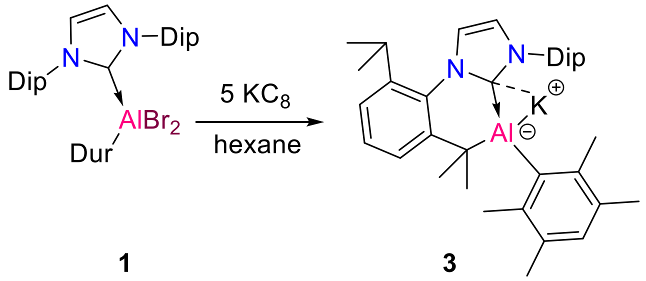

1 (0.200 g, 0.282 mmol) and freshly prepared potassium graphite (0.191 g, 1.41 mmol) were combined in a vial. The mixture was then cooled to –35 ºC and hexane (10 mL) was added. Afterward, the reaction mixture was stirred for 15 days at room temperature. The solution was filtered and the solvent was evaporated, resulting in a light red solid. The solid was subsequently extracted with 10 mL of hexane, yielding a light red solution. This hexane solution was allowed to slowly evaporate inside a glovebox, leading to the formation of a few colourless crystals of compound **3** along with free carbene. Similar to other alkyl-based Al^{I} anions,[5] compound **3** exhibited poor stability and decomposed to unidentified species during its formation, which accounted for its low yield.

Comments: We only observed a few crystals of compound 3, which appeared along with crystals of free carbene. Attempts to record HRMS data for 3 showed only signals corresponding to the free carbene.

Synthesis 5

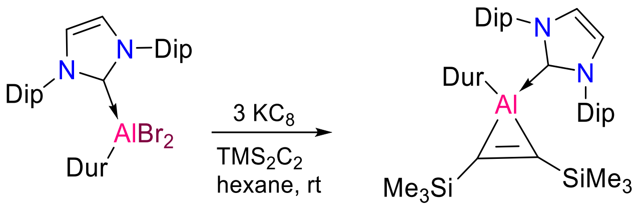

1 **5**

Bis(trimethylsilyl)acetylene (0.202 g, 1.10 mmol) and hexane (10 mL) were added to a dry Schlenk flask and cooled to 0 ºC. The mixture was then added to a pre-cooled (0 ºC) dry Schlenk flask containing **1** (0.280 g, 0.395 mmol) along with freshly prepared potassium graphite (0.161 g, 1.18 mmol). The reaction mixture was stirred vigorously and brought to room temperature within 1 h and stirred for another 15 days. A ^{1}H NMR spectrum of the crude reaction mixture showed full consumption of **1**. The resulting hexane solution was concentrated to around 5 mL and stored in the glovebox freezer (–35 ºC) to obtain red crystals of **5**. Yield: 75 mg (30%). ^{1}H NMR (400 MHz, C_6D_6, 298 K): δ = 0.29 (s, 18H, Si(C*H*$_3$)$_3$), 0.88 (d, 12H, ^{3}J = 6.9 Hz, CH(C*H*$_3$)$_2$, Dip), 1.24 (d, 12H, ^{3}J = 6.9 Hz CH(C*H*$_3$)$_2$, Dip), 2.09 (s, 6H, C*H*$_3$, Dur), 2.46 (s, 6H, C*H*$_3$, Dur), 2.71 (sept, 4H, ^{3}J = 6.9 Hz C*H*(CH$_3$)$_2$, Dip), 6.23 (s, 2H, *H*C=C*H*), 6.67 (s, 1H, Ar-*H*, Dur), 7.03 (s, 2H, Ar-*H*, Dip), 7.05 (s, 2H, Ar-*H*, Dip), 7.20 (t, ^{3}J = 6.9 Hz, 2H) ppm. ^{13}C{^{1}H} NMR (100 MHz, C_6D_6, 298 K, selected resonances based on 2D): δ = 2.6 (Si(*C*H$_3$)$_3$), 19.7 (*C*H$_3$, NHC^{Me_4}), 21.3 (*C*H$_3$, Mes), 23.2 (*C*H$_3$, Dur), 26.4 (N-*C*H$_3$), 27.7 (*C*H$_3$, Dur), 29.6 (*C*H(CH$_3$)$_2$, Dip), 125.1 (H*C*=*C*H, NHC), 128.5 (Ar-*C*H), 128.6 (Ar-*C*H), 128.7 (Ar-*C*$_q$), 128.9 (Ar-*C*$_q$), 129.2 (Ar-*C*$_q$), 131.1(Ar-*C*$_q$), 132.0 (Ar-*C*$_q$), 142.4 (Ar-*C*$_q$), 145.8 (Ar-*C*$_q$) ppm. UV-vis (hexane, 298 K) λ_{max} = 525 nm. IR (ATR, 20 °C) ṽ cm^{-1}: 1755 (m), 1455 (br, s), 1405 (m), 1382 (m), 1324 (w), 1247 (s), 1114 (m), 1060 (w), 1006 (w), 942 (m), 860 (s), 838 (s), 802 (s), 752 (s), 716 (s). HRMS LIFDI for [$C_{45}H_{67}AlN_2Si_2$]$^{+}$ = [M]$^{+}$: calcd. 719.1960; found 719.4672.

Comments: The compound is poorly stable in benzene and typical deuterated solvents, resulting in an additional peak being observed in the ^{1}H NMR and ^{13}C NMR spectra.

NMR spectra

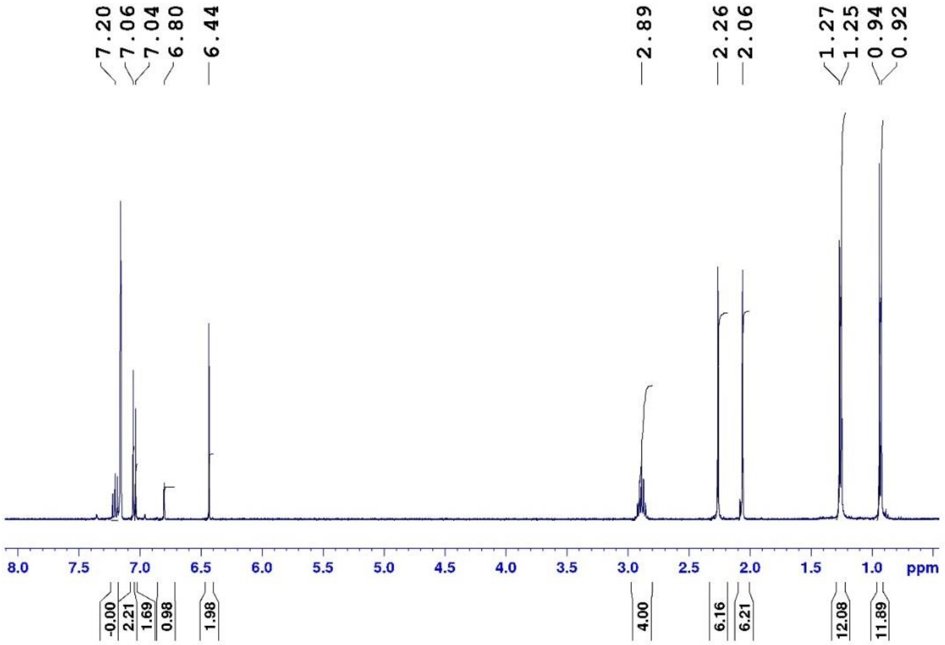

Figure S2. ^{1}H NMR spectrum of **1** in C_6D_6 at rt.

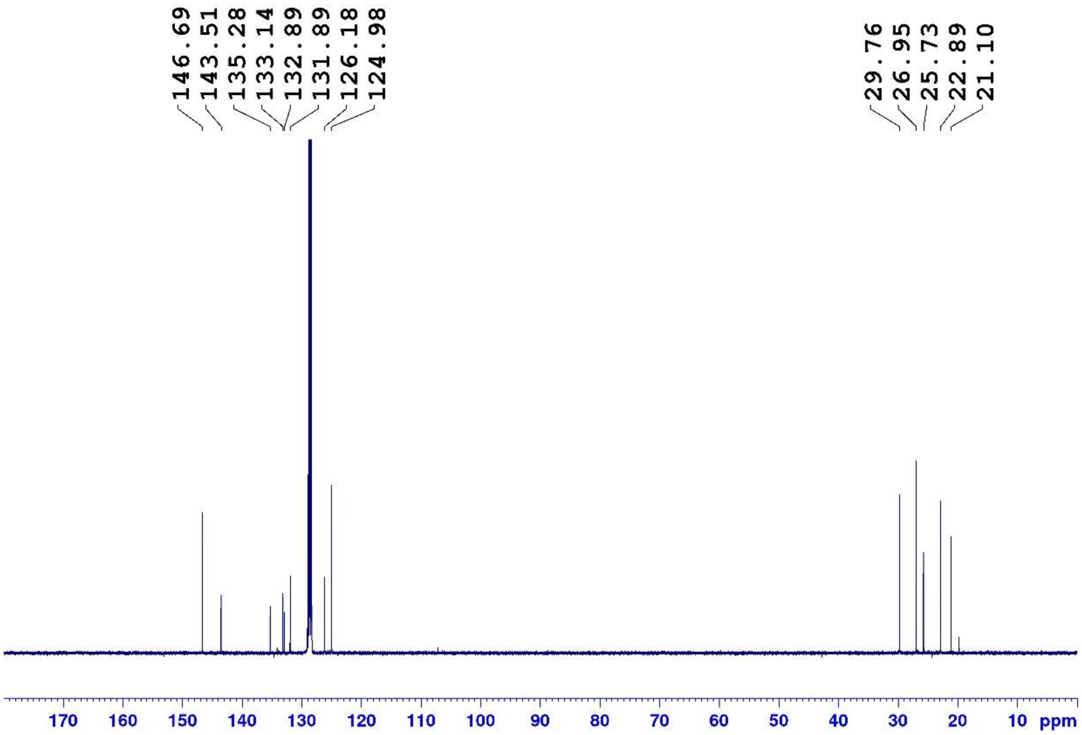

Figure S3. $^{13}C\{^{1}H\}$ NMR spectrum of **1** in C_6D_6 at rt.

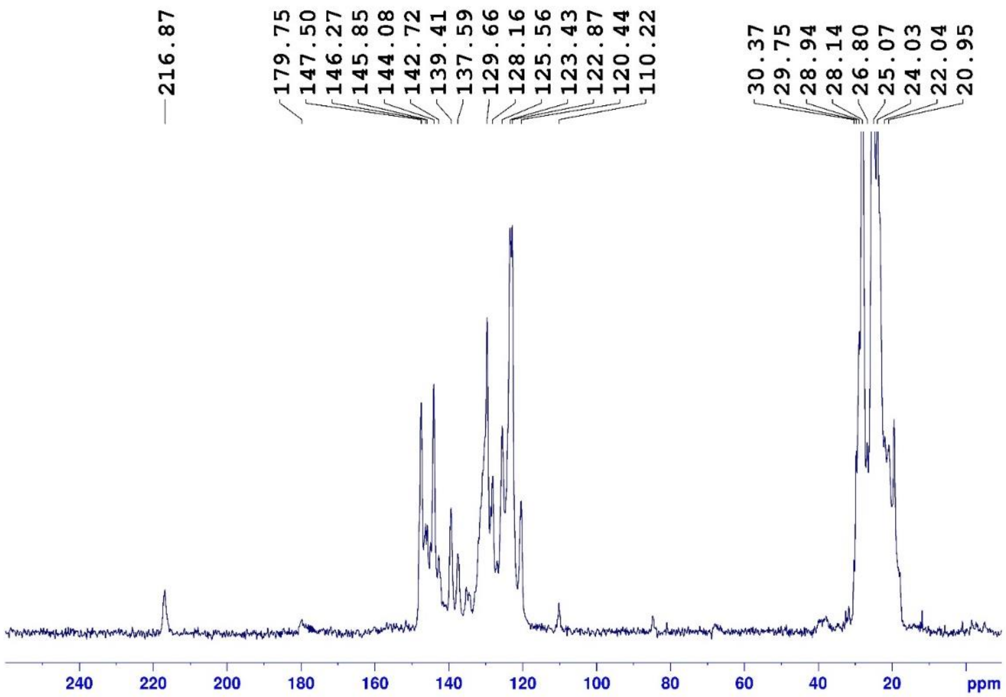

Figure S4. Solid-state ^{13}C CP/MAS NMR spectrum of **2** at 15.0 kHz

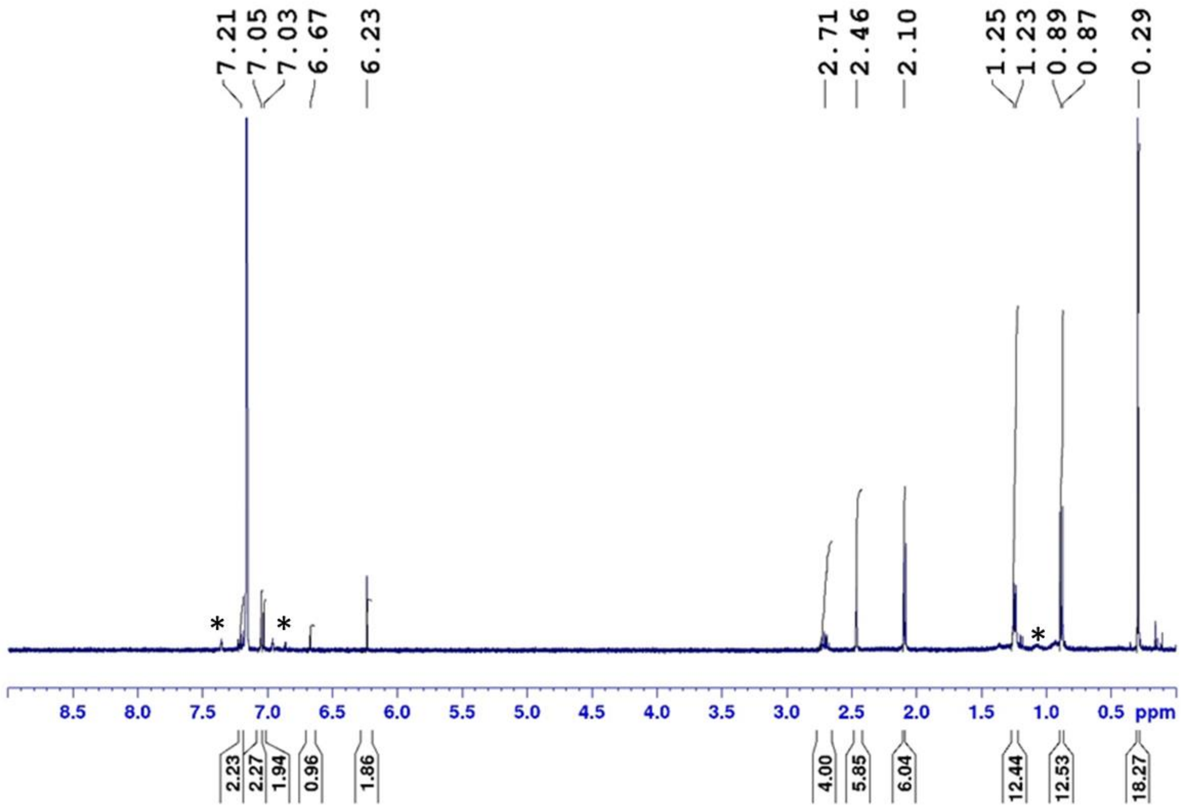

Figure S5. ^{1}H NMR spectrum of **5** in C_6D_6 at rt (* indicates unknown impurity).

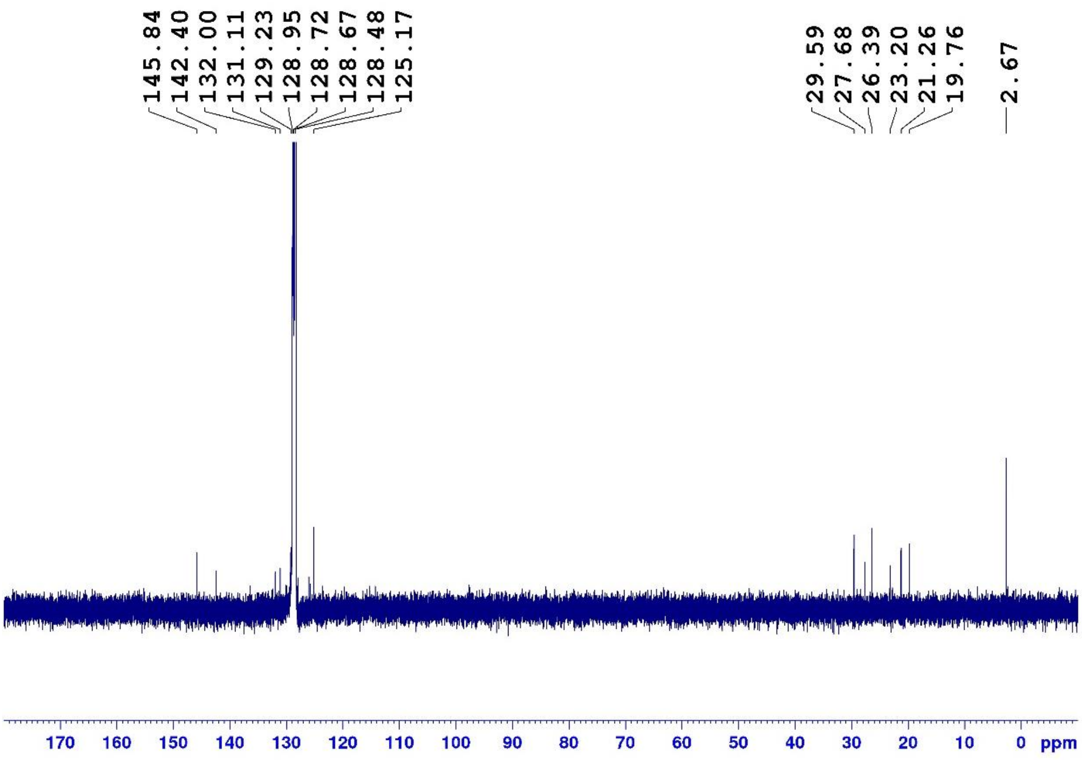

Figure S6. $^{13}C\{^{1}H\}$ NMR spectrum of 5 in C_6D_6 at rt.

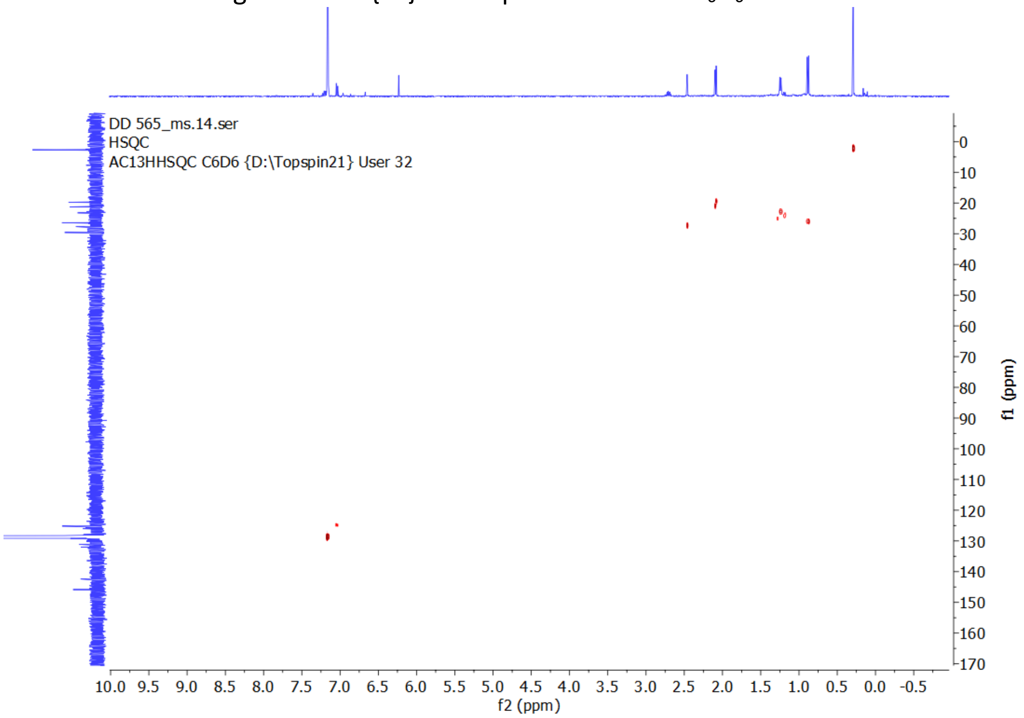

Figure S7. HSQC spectrum of **5** in C_6D_6 at rt.

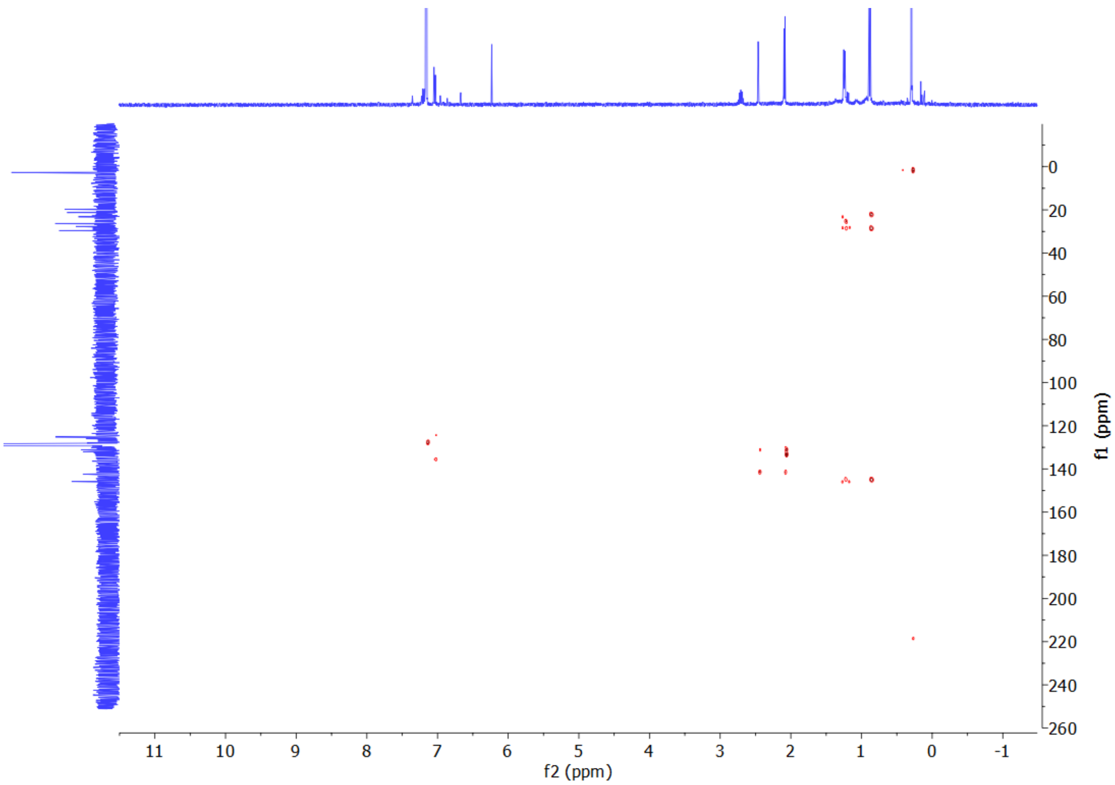

Figure S8. HMBC spectrum of **5** in C_6D_6 at rt.

UV-vis spectra

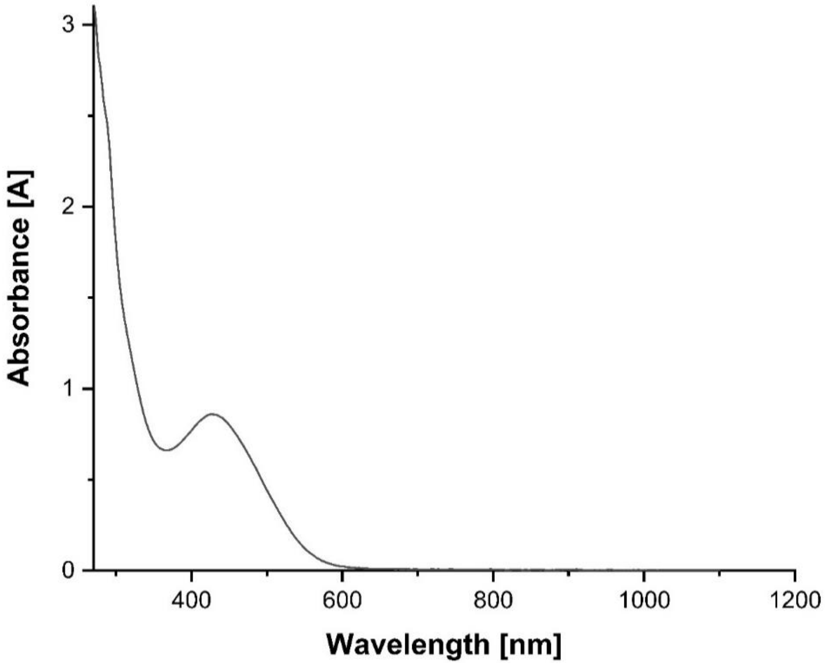

Figure S9. UV-vis spectrum of **2** in hexanes at rt.

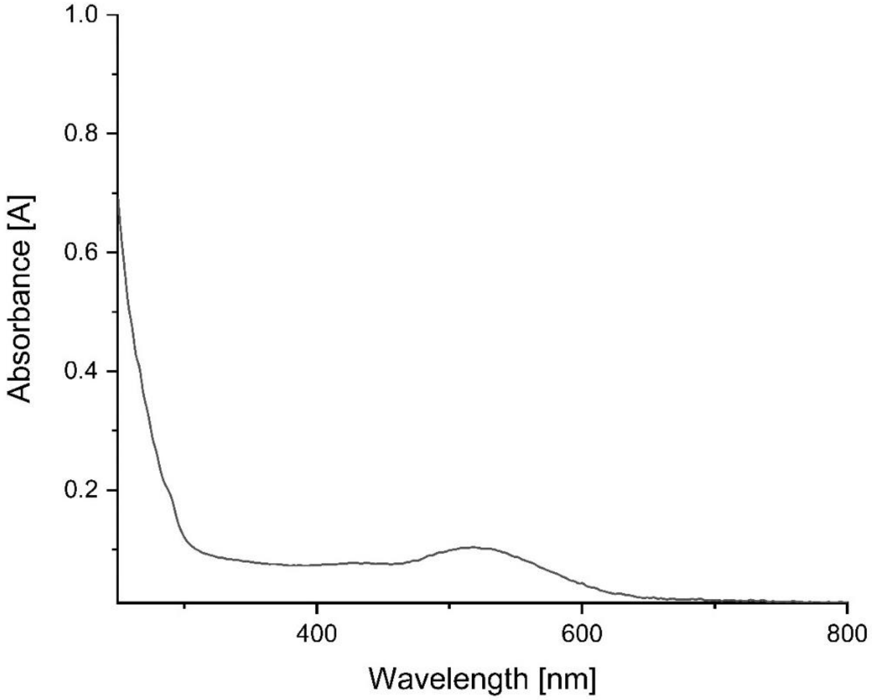

Figure S10. UV-vis spectrum of **5** in hexanes at rt.

Crystal structure determination

The crystal data of **2, 3** and **6** were collected on a BRUKER D8 QUEST diffractometer with a CMOS area detector and multi-layer mirror monochromated $Mo_{K\alpha}$ radiation. The crystal data of **1** were collected on a XTALAB SYNERGY, DUALFLEX, HYPIX diffractometer with a hybrid pixel array detector and multi-layer mirror monochromated $Cu_{K\alpha}$ radiation. The structures were solved using the intrinsic phasing method,[6] refined with the SHELXL program[7] and expanded using Fourier techniques. All non-hydrogen atoms were refined anisotropically. Hydrogen atoms were included in structure factor calculations. All hydrogen atoms were assigned to idealized geometrical positions unless otherwise stated.

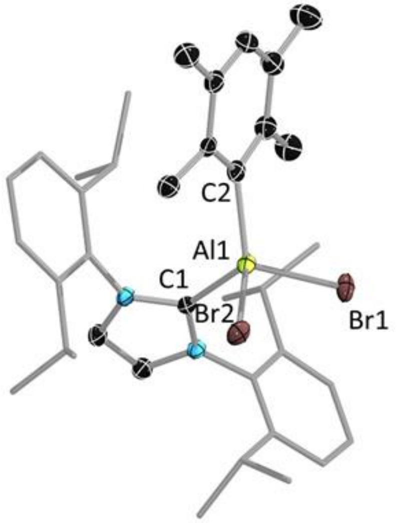

1

Figure S11. Molecular structure of **1** with thermal ellipsoids at the 50% probability level. All hydrogen atoms are omitted for clarity. Selected bond lengths [Å] and bond angles [°]: Al1-C1 2.0440(19), Al1-C2 1.9997(19), Al1-Br2 2.3335(6), Al1-Br1 2.3470(5); C1-Al1-C2 116.69(7).

Crystal data for 1 (CCDC: 2326711**)**

Empirical formula	$C_{74}H_{98}Al_2Br_4N_4$
Formula weight (g·mol^{-1})	1417.16
Temperature (K)	100(2)
Radiation, λ (Å)	$Cu_{K\alpha}$, 1.54184
Crystal system	monoclinic
Space group	$P2_1/n$
Unit cell dimensions	
a (Å)	21.7505(2)
b (Å)	16.4795(2)
c (Å)	21.8591(2)
α (°)	90
β (°)	111.9860(10)
γ (°)	90
Volume (Å^3)	7265.31(14)
Z	4
Calculated density (Mg·m^{-3})	1.296
Absorption coefficient (mm^{-1})	3.251
F(000)	2944
Theta range for collection	3.463 to 74.875°
Reflections collected	69132
Independent reflections	14240
Minimum/maximum transmission	0.63103/1.00000
Refinement method	Full-matrix least-squares on F^2
Data / parameters / restraints	14240 / 782 / 0
Goodness-of-fit on F^2	1.055
Final R indices [I>2σ(I)]	R_1 = 0.0293, wR_2 = 0.0765
R indices (all data)	R_1 = 0.0320, wR_2 = 0.0780
Maximum/minimum residual electron density (e·Å^{-3})	0.881 / –0.545

Crystal data for 2 (CCDC: 2326709**)**

Empirical formula	$C_{84}H_{111}Al_3N_4$
Formula weight (g·mol^{-1})	1257.70
Temperature (K)	100(2)
Radiation, λ (Å)	$Mo_{K\alpha}$, 0.71073
Crystal system	trigonal
Space group	**R**-3
Unit cell dimensions	
a (Å)	37.395(4)
b (Å)	37.395(4)
c (Å)	36.972(4)
α (°)	90
β (°)	90
γ (°)	120
Volume (Å^3)	44774(10)
Z	18
Calculated density (Mg·m^{-3})	0.840
Absorption coefficient (mm^{-1})	0.072
F(000)	12276
Theta range for collection	1.373 to 26.500°
Reflections collected	624461
Independent reflections	20563
Minimum/maximum transmission	0.7090/0.7447
Refinement method	Full-matrix least-squares on F^2
Data / parameters / restraints	20563 / 848 / 0
Goodness-of-fit on F^2	1.062
Final R indices [I>2σ(I)]	R_1 = 0.0499, wR_2 = 0.1474
R indices (all data)	R_1 = 0.0607, wR_2 = 0.1629
Maximum/minimum residual electron density (e·Å^{-3})	0.356 / –0.267

Refinement special details: The unit cell contains co-crystallized hexane which have been treated as a diffuse contribution to the overall scattering without specific atom positions by SQUEEZE/PLATON[8]

Crystal data for 3 (CCDC: 2326710**)**

Empirical formula	$C_{37}H_{48}AlKN_2$
Formula weight (g·mol^{-1})	586.85
Temperature (K)	100(2)
Radiation, λ (Å)	$Mo_{K\alpha}$, 0.71073
Crystal system	monoclinic
Space group	$P2_1/n$
Unit cell dimensions	
a (Å)	15.153(5)
b (Å)	14.835(2)
c (Å)	16.200(5)
α (°)	90
β (°)	113.636(17)
γ (°)	90
Volume (Å^3)	3336.2(16)
Z	4
Calculated density (Mg·m^{-3})	1.168
Absorption coefficient (mm^{-1})	0.213
F(000)	1264
Theta range for collection	2.745 to 27.513°
Reflections collected	50737
Independent reflections	7637
Minimum/maximum transmission	0.6803/0.7456
Refinement method	Full-matrix least-squares on F^2
Data / parameters / restraints	7637 / 382 / 0
Goodness-of-fit on F^2	1.060
Final R indices [I>2σ(I)]	R_1 = 0.0440, wR_2 = 0.1204
R indices (all data)	R_1 = 0.0481, wR_2 = 0.1230
Maximum/minimum residual electron density (e·Å^{-3})	0.676 / –0.578

Crystal data for 5 (CCDC: 2326719**)**

Empirical formula	$C_{45}H_{67}AlN_2Si_2$
Formula weight (g·mol^{-1})	719.16
Temperature (K)	109(2)
Radiation, λ (Å)	$Mo_{K\alpha}$, 0.71073
Crystal system	triclinic
Space group	$P\bar{1}$
Unit cell dimensions	
a (Å)	9.5531(18)
b (Å)	12.491(3)
c (Å)	19.730(2)
α (°)	90.074(10)
β (°)	102.911(10)
γ (°)	107.935(19)
Volume (Å^3)	2176.9(8)
Z	2
Calculated density (Mg·m^{-3})	1.097
Absorption coefficient (mm^{-1})	0.133
F(000)	784
Theta range for collection	2.453 to 26.485°
Reflections collected	43434
Independent reflections	8907
Minimum/maximum transmission	0.7003/0.7454
Refinement method	Full-matrix least-squares on F^2
Data / parameters / restraints	8907 / 469 / 0
Goodness-of-fit on F^2	1.051
Final R indices [I>2σ(I)]	R_1 = 0.0569, wR_2 = 0.1530
R indices (all data)	R_1 = 0.0625, wR_2 = 0.1578
Maximum/minimum residual electron density (e·Å^{-3})	1.420 / –0.485

Computational Details

All calculations were performed using the Gaussian 16, Rev C.01[9] and the ORCA 5.0.4[10] quantum chemistry program packages. Geometry optimizations were performed at the ωB97X-D[11]/Def2-SVP[12] level of theory. Vertical and adiabatic singlet-triplet gaps were obtained from single-point calculations at the (U)ωB97X-D/Def2-TZVP[12] level of theory using the appropriate geometries. All optimized geometries were characterized as minima on the corresponding potential energy surfaces by vibrational calculations, which revealed that all eigenvalues in the Hessian matrices are positive. GaussView 6.0.16[13] was used for graphical representation of the molecular orbitals. For the estimation of free energy values in solution, thermal corrections to the Gibbs free energy, obtained from frequency calculations at the ωB97X-D/Def2-SVP level, were added to single-point energy calculations at the ωB97X-D/Def2-TZVP level, incorporating solvent effects using the integral equation formalism polarizable continuum model (IEFPCM)[14] with n-hexane (ε = 1.8819) as the solvent. To accurately account for associative and dissociative steps, a concentration correction of $\Delta G^{o\rightarrow *}$ = 1.89 kcal/mol was applied to all calculated species, reflecting the change in the standard state from the gas phase (1 atm) to the condensed phase (1 M).[15-17]

To investigate the different bonding situations, calculations based on the intrinsic bond orbital (IBO)[18] method were performed using the IBOView software, version v20211019-RevA.[18,19] The bonding situations were also evaluated by Mayer bond order (MBO)[20] analysis and charge decomposition analysis.[21]

Finally, to analyze the oxidation states of the aluminum atoms of **2** calculations of the localized orbital bonding analysis (LOBA)[22] were performed. The calculations of the MBOs and LOBA were done in Multiwfn 3.8.[23]

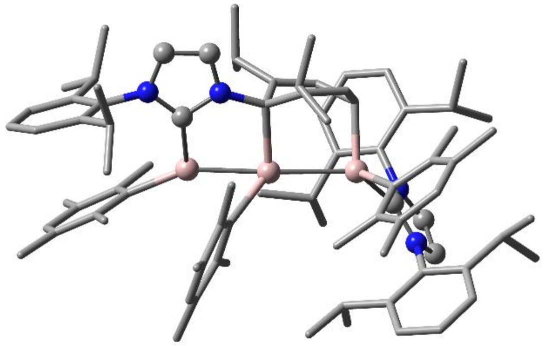

9

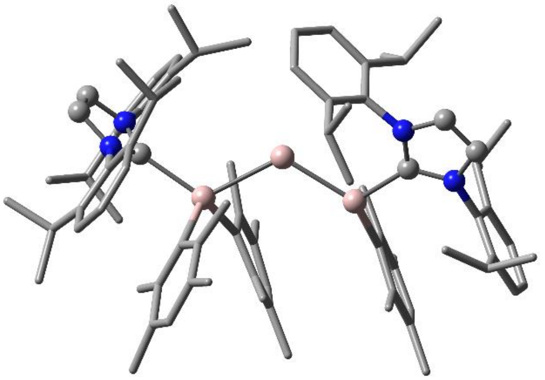

9’

Figure S12. Comparison between the optimized structures of intermediates **9** and **9’**.

Cartesian Coordinates

Compound 2, ωB97X-D/Def2-SVP

Geometry of the ground state

Energy = –4210.53797073 E_h

Al	2.452415000	-0.020652000	-0.510374000
Al	-2.491240000	0.230352000	0.708875000
Al	-0.063366000	-0.251743000	0.016075000
N	-2.060354000	-0.133794000	-2.260599000
N	-4.193642000	-0.246151000	-2.009215000
N	4.703794000	-0.526624000	1.543024000
N	4.496783000	-2.285065000	0.336806000
C	-2.600712000	-0.323620000	-3.514512000
H	-1.971894000	-0.410172000	-4.395365000
C	-3.943088000	-0.392908000	-3.361658000
H	-4.744819000	-0.545096000	-4.077555000
C	-5.898771000	-1.520264000	-0.793433000
C	3.161957000	1.855063000	-0.807410000
C	-3.801865000	-0.668865000	1.961364000
C	4.042378000	2.113286000	-1.884063000
C	-3.031655000	-0.067916000	-1.319649000
C	-7.213371000	-1.602153000	-0.330009000
H	-7.530508000	-2.480231000	0.232060000
C	-8.132455000	-0.588070000	-0.579489000

H	-9.158008000	-0.682888000	-0.215867000
C	-5.527690000	-0.347233000	-1.481530000
C	-3.494037000	-1.808990000	2.742036000
C	1.091845000	-2.024963000	-2.103999000
H	1.419271000	-3.068350000	-2.090850000
C	-7.749460000	0.539657000	-1.291590000
H	-8.479044000	1.328982000	-1.484987000
C	-0.620543000	-0.269258000	-2.001640000
C	3.973542000	-1.045306000	0.523444000
C	5.528691000	-2.533412000	1.222909000
H	6.049945000	-3.485131000	1.243931000
C	5.654593000	-1.424962000	1.987090000
H	6.313423000	-1.196972000	2.818517000
C	4.833146000	0.998467000	-2.525745000
H	4.540816000	0.815281000	-3.573636000
H	4.701319000	0.056336000	-1.981611000
H	5.912397000	1.226526000	-2.534458000
C	2.109037000	-0.966497000	-2.319136000
H	3.013493000	-1.376840000	-2.783282000
C	-6.436017000	0.691309000	-1.746604000
C	1.518872000	0.154192000	-3.112044000
H	2.189043000	0.754193000	-3.732742000
C	-4.941024000	-2.691692000	-0.621999000
H	-3.952648000	-2.278283000	-0.372521000

C	-0.230958000	-1.746140000	-2.008643000
C	-5.054679000	-0.043965000	2.166588000
C	-5.647102000	-1.690030000	3.826606000
H	-6.367732000	-2.092080000	4.546543000
C	3.912878000	-3.343346000	-0.443436000
C	-1.270939000	-2.864802000	-2.010307000
H	-2.263623000	-2.415933000	-1.881516000
C	-2.207706000	2.204220000	1.155080000
C	-5.324260000	-3.632574000	0.518302000
H	-5.522152000	-3.083387000	1.447766000
H	-4.496443000	-4.331848000	0.710587000
H	-6.210236000	-4.238437000	0.267501000
C	0.238618000	0.534586000	-2.954197000
C	6.396365000	3.238765000	0.260449000
H	6.934626000	3.939976000	0.918569000
H	5.419962000	3.671367000	0.008700000
H	6.978995000	3.156056000	-0.669486000
C	-1.104171000	-3.856573000	-0.861371000
H	-0.117667000	-4.342774000	-0.893850000
H	-1.872206000	-4.645525000	-0.912397000
H	-1.202392000	-3.351534000	0.108522000
C	3.581274000	4.473458000	-1.745729000
H	3.723085000	5.490420000	-2.125952000
C	-1.279744000	-3.592862000	-3.360038000

H	-1.405149000	-2.887852000	-4.194640000
H	-2.098681000	-4.328769000	-3.407435000
H	-0.329310000	-4.127333000	-3.518395000
C	4.235983000	3.416650000	-2.376498000
C	-4.820395000	-3.480102000	-1.934665000
H	-4.454932000	-2.861678000	-2.766027000
H	-5.798146000	-3.897366000	-2.223640000
H	-4.116408000	-4.317232000	-1.811226000
C	-4.413337000	-2.319992000	3.678753000
C	-2.266659000	3.291485000	0.253917000
C	-5.986834000	-0.558309000	3.088147000
C	4.346649000	-3.560073000	-1.761740000
C	2.978505000	-4.170696000	0.202239000
C	5.473681000	-2.739223000	-2.366601000
H	5.463934000	-1.757938000	-1.871970000
C	-1.793577000	1.366137000	3.512539000
H	-1.128422000	1.549300000	4.365292000
H	-1.523584000	0.383305000	3.100006000
H	-2.818861000	1.261916000	3.903117000
C	-6.054445000	1.955796000	-2.501438000
H	-4.969448000	1.941513000	-2.671050000
C	2.557430000	2.960796000	-0.166238000
C	-1.728720000	2.457830000	2.468355000
C	4.427264000	0.690871000	2.263293000

C	2.588344000	-0.601377000	3.494272000
H	2.562816000	-1.167573000	2.553427000
C	3.756381000	-4.613342000	-2.463360000
H	4.051053000	-4.804398000	-3.496282000
C	3.431863000	0.642631000	3.255385000
C	5.194896000	1.836241000	1.999974000
C	1.626619000	2.781154000	1.006129000
H	1.828062000	3.508650000	1.806070000
H	1.712269000	1.782681000	1.448767000
H	0.571282000	2.927298000	0.715638000
C	2.428566000	-5.219389000	-0.540624000
H	1.695662000	-5.880855000	-0.073327000
C	-0.270109000	1.841705000	-3.548890000
H	-1.364515000	1.875154000	-3.436027000
C	6.252036000	1.860701000	0.907618000
H	5.915889000	1.163723000	0.124438000
C	2.801314000	-5.429723000	-1.862758000
H	2.350424000	-6.245567000	-2.431513000
C	5.329477000	-2.496090000	-3.869739000
H	6.086285000	-1.770738000	-4.203259000
H	4.339551000	-2.093758000	-4.127297000
H	5.485944000	-3.418797000	-4.450194000
C	7.614785000	1.383839000	1.429672000
H	8.365866000	1.426666000	0.625944000

H	7.588062000	0.350943000	1.801784000
H	7.961705000	2.030460000	2.251295000
C	-2.173183000	-2.528431000	2.615564000
H	-1.500330000	-2.012917000	1.918601000
H	-2.307279000	-3.564443000	2.258910000
H	-1.656232000	-2.598509000	3.587592000
C	-6.730648000	2.003194000	-3.876673000
H	-6.411621000	2.897448000	-4.433620000
H	-7.827341000	2.039496000	-3.780613000
H	-6.480541000	1.119152000	-4.482122000
C	4.951607000	2.960838000	2.792317000
H	5.513984000	3.877337000	2.610979000
C	2.608803000	-3.994926000	1.668362000
H	3.174690000	-3.141442000	2.063317000
C	-5.427179000	1.229325000	1.446289000
H	-5.372507000	2.095451000	2.127832000
H	-6.449489000	1.188774000	1.044733000
H	-4.754585000	1.454365000	0.610872000
C	-4.094135000	-3.545727000	4.495507000
H	-3.199586000	-3.398896000	5.122603000
H	-3.887764000	-4.418043000	3.853251000
H	-4.928565000	-3.809824000	5.160620000
C	2.765817000	4.273592000	-0.633094000
C	-1.768966000	4.561800000	0.608641000

C	3.995247000	2.935849000	3.801107000
H	3.823016000	3.827351000	4.407923000
C	6.827162000	-3.396095000	-2.059153000
H	7.651276000	-2.788927000	-2.463651000
H	6.885141000	-4.397662000	-2.513919000
H	6.992527000	-3.508314000	-0.978204000
C	1.129328000	-3.666723000	1.865963000
H	0.845527000	-2.771445000	1.292886000
H	0.921441000	-3.469338000	2.928689000
H	0.478030000	-4.494426000	1.546888000
C	-2.896618000	3.150863000	-1.109518000
H	-3.550729000	4.004493000	-1.340496000
H	-3.508579000	2.246405000	-1.166839000
H	-2.148892000	3.097634000	-1.913994000
C	-1.245807000	3.726086000	2.836874000
C	5.149399000	3.681494000	-3.545223000
H	5.124095000	4.740431000	-3.838864000
H	4.871223000	3.081459000	-4.426565000
H	6.196630000	3.427053000	-3.308485000
C	3.240769000	1.791169000	4.026802000
H	2.476141000	1.795868000	4.804928000
C	-7.329107000	0.096885000	3.289493000
H	-7.227858000	1.152427000	3.588672000
H	-7.911428000	-0.418481000	4.066648000

H	-7.922133000	0.086680000	2.359729000
C	3.032924000	-5.218192000	2.488512000
H	4.106821000	-5.428934000	2.370434000
H	2.478633000	-6.120270000	2.185601000
H	2.833838000	-5.047974000	3.557559000
C	-6.366214000	3.216479000	-1.688072000
H	-5.994873000	4.109461000	-2.213603000
H	-5.890195000	3.182136000	-0.698139000
H	-7.449607000	3.346718000	-1.540139000
C	-1.255644000	4.749497000	1.888643000
H	-0.856629000	5.731921000	2.161782000
C	1.135145000	-0.262748000	3.812222000
H	1.015230000	0.181558000	4.812717000
H	0.514265000	-1.170269000	3.780047000
H	0.728190000	0.439084000	3.072055000
C	3.204091000	-1.504040000	4.569234000
H	4.225965000	-1.815905000	4.307228000
H	2.597279000	-2.414189000	4.697782000
H	3.246650000	-0.984836000	5.539820000
C	2.085431000	5.445444000	0.025188000
H	2.282591000	6.379835000	-0.519431000
H	2.427913000	5.581444000	1.064509000
H	0.995677000	5.298582000	0.074085000
C	-1.780235000	5.706730000	-0.372828000

H	-1.266776000	6.587368000	0.039004000
H	-2.807254000	6.013215000	-0.632682000
H	-1.279925000	5.435540000	-1.316299000
C	0.299122000	3.036153000	-2.773500000
H	1.391486000	3.091849000	-2.890677000
H	0.093740000	2.948288000	-1.695694000
H	-0.135888000	3.985469000	-3.128840000
C	-0.694760000	4.006306000	4.214373000
H	-0.375109000	5.054608000	4.302146000
H	0.181921000	3.374189000	4.434337000
H	-1.437380000	3.817098000	5.005550000
C	0.024560000	1.972234000	-5.044900000
H	-0.403763000	2.904410000	-5.446483000
H	-0.392579000	1.127879000	-5.615526000
H	1.108125000	1.998119000	-5.238187000

Compound 3, ωB97X-D/Def2-SVP

Geometry of the ground state

Energy = –2389.02830306E$_h$

K	-3.095964000	-0.580789000	0.095161000
Al	-0.241497000	-0.631079000	-1.513060000
N	0.112651000	2.078936000	0.033301000
N	1.964015000	1.087452000	-0.406400000
C	0.626080000	0.854313000	-0.284436000

C	-1.278159000	2.303165000	0.257838000
C	-0.675858000	-2.285200000	-0.325166000
C	-0.421807000	-2.352952000	1.073906000
C	-1.793543000	2.082781000	1.546280000
C	1.094264000	3.052972000	0.059071000
H	0.875776000	4.093364000	0.278777000
C	2.913322000	0.028705000	-0.583157000
C	0.819941000	-1.728389000	1.668459000
H	0.606542000	-0.843578000	2.291854000
H	1.518796000	-1.426529000	0.882660000
H	1.344106000	-2.450345000	2.315996000
C	-2.534476000	-3.885242000	0.020166000
C	1.543524000	-0.756565000	-2.597037000
C	-2.082569000	2.719103000	-0.822497000
C	-3.982299000	2.709544000	0.698347000
H	-5.046433000	2.885229000	0.874187000
C	-2.346773000	-3.786395000	1.403822000
H	-3.003860000	-4.350079000	2.074685000
C	1.432567000	-1.933122000	-3.569018000
H	1.281529000	-2.894839000	-3.051911000
H	2.321975000	-2.037665000	-4.222677000
H	0.568679000	-1.784447000	-4.237573000
C	-1.691168000	-3.147680000	-0.831902000
C	-3.444000000	2.924867000	-0.568595000

H	-4.094390000	3.257502000	-1.379381000
C	3.927794000	-0.130301000	0.378765000
C	4.801692000	-1.212269000	0.237393000
H	5.609920000	-1.353486000	0.958229000
C	3.571897000	-1.993337000	-1.684509000
H	3.447094000	-2.743660000	-2.463752000
C	2.260328000	2.428899000	-0.239650000
H	3.265897000	2.821685000	-0.349848000
C	1.691974000	0.530078000	-3.431646000
H	1.725703000	1.449949000	-2.827306000
H	0.837857000	0.634960000	-4.120413000
H	2.617042000	0.509682000	-4.040708000
C	-3.166186000	2.292902000	1.747629000
H	-3.595489000	2.155773000	2.742446000
C	-1.272810000	-3.072572000	1.942052000
C	-0.893382000	1.685109000	2.704290000
H	0.038891000	1.290904000	2.276049000
C	4.619033000	-2.135802000	-0.784230000
H	5.297600000	-2.987522000	-0.875222000
C	2.681834000	-0.904687000	-1.630547000
C	-0.994201000	-3.163723000	3.422686000
H	-1.847792000	-3.596631000	3.964315000
H	-0.764408000	-2.186839000	3.871856000
H	-0.120055000	-3.808346000	3.614640000

C	-1.892322000	-3.291522000	-2.322102000
H	-1.615306000	-4.308115000	-2.648823000
H	-1.276766000	-2.578059000	-2.885937000
H	-2.938701000	-3.130545000	-2.632558000
C	-1.487573000	3.040046000	-2.182842000
H	-0.533288000	2.498591000	-2.254322000
C	4.071385000	0.763196000	1.606301000
H	3.202242000	1.433093000	1.654538000
C	-3.617601000	-4.781267000	-0.528792000
H	-4.182417000	-5.267604000	0.279510000
H	-3.195043000	-5.574065000	-1.166435000
H	-4.336144000	-4.230392000	-1.158538000
C	-1.494593000	0.574868000	3.566807000
H	-0.768857000	0.253548000	4.328853000
H	-1.746568000	-0.311668000	2.963332000
H	-2.400607000	0.901964000	4.100453000
C	4.048741000	-0.050794000	2.905652000
H	4.929356000	-0.704678000	2.997292000
H	3.152565000	-0.685769000	2.956514000
H	4.045517000	0.622371000	3.777551000
C	5.327249000	1.637620000	1.523255000
H	5.397677000	2.310401000	2.392416000
H	5.333924000	2.253568000	0.610917000
H	6.237178000	1.017423000	1.502171000

C	-1.209504000	4.546078000	-2.289901000
H	-0.551060000	4.900500000	-1.483019000
H	-2.144510000	5.127008000	-2.238160000
H	-0.720692000	4.775918000	-3.248658000
C	-2.349949000	2.549858000	-3.344495000
H	-2.517381000	1.465766000	-3.247895000
H	-1.824087000	2.726408000	-4.294966000
H	-3.316592000	3.076612000	-3.403773000
C	-0.531834000	2.916428000	3.543757000
H	-1.430350000	3.364946000	3.997066000
H	-0.044700000	3.686800000	2.927954000
H	0.159624000	2.642908000	4.355305000

Compound 4', ωB97X-D/Def2-SVP

Geometry of the ground state

Energy = –1789.76074655 E_h

C	0.7279020	-0.3900990	0.2184450
C	2.2134750	-1.1328200	1.7650830
H	3.1839470	-1.2295000	2.2415330
C	1.0143680	-1.6862950	2.0591670
N	0.1215940	-1.2121580	1.1132180
C	-1.2597180	-1.5974330	1.0803390
C	-2.2247920	-0.6985080	1.5719070
C	-3.5476970	-1.1424630	1.6023420

H	-4.3309790	-0.4774650	1.9668720
C	-3.8869860	-2.4234040	1.1720670
H	-4.9281360	-2.7505180	1.2159850
C	-2.9151830	-3.2770500	0.6671280
H	-3.1995190	-4.2697570	0.3110910
C	-1.5769950	-2.8757650	0.5958700
C	-0.5226400	-3.8154820	0.0285210
H	0.4252490	-3.2611230	-0.0387090
C	-0.8651410	-4.2696550	-1.3934400
H	-0.0642180	-4.9131060	-1.7882460
H	-0.9640170	-3.4077920	-2.0695500
H	-1.8026760	-4.8464280	-1.4260890
C	-0.2937660	-5.0105660	0.9609170
H	0.5085690	-5.6566260	0.5730340
H	-1.2043570	-5.6235640	1.0532570
H	-0.0088260	-4.6846300	1.9727100
C	-1.8312960	0.6868530	2.0646040
H	-1.0596360	1.0557070	1.3717430
C	-1.2251070	0.6285080	3.4738860
H	-0.9301880	1.6372180	3.8024940
H	-0.3312270	-0.0094550	3.5192340
H	-1.9591300	0.2386780	4.1971930
C	-2.9810020	1.6902960	2.0170080
H	-2.5959810	2.7044070	2.2022220

H	-3.7386600	1.4840020	2.7906550
H	-3.4681270	1.6949090	1.0327950
Al	0.0364470	-0.0914220	-1.8063940
C	-1.6164200	1.0560830	-1.4595580
C	-2.9077670	0.5614290	-1.7394250
C	-4.0358090	1.4015950	-1.6773320
C	-3.8553000	2.7477130	-1.3574940
H	-4.7296330	3.4053810	-1.3116880
C	-2.5946340	3.2763470	-1.0781960
C	-1.4811180	2.4192160	-1.1265350
C	-0.1237920	2.9858900	-0.7893340
H	0.1368560	3.8527170	-1.4183570
H	0.6865060	2.2477750	-0.9101850
H	-0.0877120	3.3333830	0.2570850
C	-3.1120030	-0.8863390	-2.1155210
H	-3.6475680	-0.9894930	-3.0732920
H	-3.6994180	-1.4249960	-1.3557800
H	-2.1552860	-1.4210280	-2.2220880
C	-5.4174240	0.8630250	-1.9459510
H	-6.1833370	1.6410810	-1.8176460
H	-5.6632220	0.0304460	-1.2663000
H	-5.5081220	0.4687700	-2.9713470
C	-2.4439430	4.7292330	-0.7078020
H	-1.7838050	5.2635060	-1.4105720

H	-1.9986790	4.8479080	0.2942480
H	-3.4149980	5.2442710	-0.7044110
C	3.0741410	0.3665490	-0.0198880
N	2.0182540	-0.3400660	0.6474960
C	3.3520780	1.6922460	0.3606160
C	3.8034690	-0.3141820	-1.0105210
C	4.4027810	2.3400980	-0.2941240
C	2.5768110	2.3808650	1.4754680
H	4.6504840	3.3695440	-0.0318500
C	5.1401650	1.6921090	-1.2799280
H	5.9558620	2.2183610	-1.7804000
C	4.8446150	0.3808840	-1.6320450
H	5.4326300	-0.1135740	-2.4071990
C	3.5260320	-1.7686250	-1.3590440
H	2.5083560	-2.0055360	-1.0149260
C	4.5042070	-2.6884270	-0.6167130
C	3.5422620	-2.0315800	-2.8652310
H	4.2777950	-3.7446110	-0.8294700
H	4.4518960	-2.5441500	0.4729970
H	5.5425480	-2.4944450	-0.9299940
H	3.2350230	-3.0693070	-3.0654170
H	4.5454870	-1.8976240	-3.3001350
H	2.8328560	-1.3657450	-3.3781370
H	1.5526470	1.9760840	1.4590150

C	2.4784090	3.8970480	1.2924180
C	3.1843420	2.0590870	2.8483040
H	1.7486070	4.3146690	2.0022450
H	2.1587930	4.1677620	0.2766380
H	3.4413490	4.3934130	1.4909840
H	2.6103880	2.5523010	3.6478750
H	4.2246000	2.4170020	2.9041500
H	3.1861420	0.9809910	3.0574340
H	0.7089620	-2.3640470	2.8503830

Compound CAAC-4', ωB97X-D/Def2-SVP

Geometry of the ground state

Energy = –1465.56163577 E_h

C	-0.759253000	-1.377517000	-0.499282000
C	-1.121185000	-2.859322000	-0.382084000
C	-2.613142000	-2.832541000	0.004289000
H	-3.220315000	-2.920329000	-0.909727000
H	-2.894176000	-3.665862000	0.665257000
C	-2.877790000	-1.469709000	0.659253000
N	-1.704028000	-0.681235000	0.143402000
C	-1.542066000	0.724927000	0.418629000
C	-0.791261000	1.115357000	1.554789000
C	-0.687343000	2.478214000	1.844916000
H	-0.118219000	2.793767000	2.721990000

C	-1.282796000	3.437584000	1.036899000
H	-1.193502000	4.497465000	1.284463000
C	-1.967606000	3.042799000	-0.102635000
H	-2.403890000	3.803839000	-0.753191000
C	-2.106045000	1.693154000	-0.446666000
C	-2.838234000	1.371043000	-1.746446000
H	-2.837141000	0.279082000	-1.874076000
C	-2.124300000	1.960215000	-2.970627000
H	-2.687867000	1.716735000	-3.884238000
H	-1.115989000	1.540254000	-3.092400000
H	-2.045875000	3.056872000	-2.906563000
C	-4.288934000	1.875920000	-1.727218000
H	-4.834474000	1.499077000	-2.605958000
H	-4.321705000	2.975839000	-1.770557000
H	-4.836210000	1.566548000	-0.827773000
C	-0.073826000	0.135105000	2.474277000
H	-0.341765000	-0.877225000	2.155764000
C	-0.496820000	0.298116000	3.939516000
H	-0.074745000	-0.516570000	4.547901000
H	-1.588747000	0.286227000	4.065430000
H	-0.123865000	1.244039000	4.362202000
C	1.448994000	0.246681000	2.344011000
H	1.939203000	-0.466964000	3.024386000
H	1.802595000	1.256288000	2.607900000

H	1.781846000	0.030016000	1.322002000
C	-0.905256000	-3.673089000	-1.661475000
H	-1.245358000	-4.709817000	-1.506057000
H	0.157970000	-3.714991000	-1.942165000
H	-1.457114000	-3.241859000	-2.508602000
C	-0.248534000	-3.462735000	0.737616000
H	-0.520413000	-4.518182000	0.896441000
H	-0.368642000	-2.935448000	1.693345000
H	0.814768000	-3.415901000	0.467956000
C	-2.892974000	-1.533571000	2.190152000
H	-3.779202000	-2.096313000	2.517148000
H	-2.959976000	-0.524071000	2.619228000
H	-2.009485000	-2.031434000	2.606653000
C	-4.214720000	-0.885754000	0.210824000
H	-5.026544000	-1.535447000	0.569928000
H	-4.291291000	-0.825856000	-0.881799000
H	-4.373346000	0.114996000	0.637105000
Al	0.461634000	-0.626130000	-1.933471000
C	2.222241000	-0.094817000	-1.109004000
C	2.490437000	1.264854000	-0.853524000
C	3.696714000	1.656129000	-0.244821000
C	4.631417000	0.675112000	0.085051000
H	5.567479000	0.974656000	0.567491000
C	4.414246000	-0.676488000	-0.187338000

C	3.203832000	-1.054655000	-0.793412000
C	2.971852000	-2.514550000	-1.098192000
H	3.747780000	-2.921657000	-1.766708000
H	2.006018000	-2.681615000	-1.602109000
H	2.981042000	-3.136778000	-0.187741000
C	1.473188000	2.327994000	-1.184861000
H	1.898781000	3.123290000	-1.817628000
H	1.078853000	2.811102000	-0.277164000
H	0.606110000	1.920089000	-1.727495000
C	3.969400000	3.104041000	0.070211000
H	4.939540000	3.229485000	0.571345000
H	3.191719000	3.525346000	0.728358000
H	3.979621000	3.723364000	-0.841457000
C	5.458760000	-1.702692000	0.169390000
H	5.816689000	-2.243486000	-0.722043000
H	5.062244000	-2.463056000	0.862071000
H	6.330589000	-1.235985000	0.648972000

Compound 5, ωB97X-D/Def2-SVP

Geometry of the ground state

Energy = –2684.05097709E_h

Al	0.022490000	0.183560000	0.734050000
Si	3.174245000	-0.353133000	2.513193000
Si	1.759663000	3.308007000	1.475943000

N	0.896247000	-0.716296000	-2.063113000
N	-1.131250000	-0.024433000	-2.119031000
C	0.501812000	-0.751172000	-3.387214000
H	1.164740000	-1.100842000	-4.171971000
C	-0.100204000	-0.255290000	-1.268403000
C	-0.777587000	-0.316247000	-3.422648000
H	-1.478439000	-0.208825000	-4.243885000
C	-1.626252000	-0.301454000	1.740172000
C	-2.654159000	0.636882000	1.978409000
C	-3.843592000	-0.977739000	3.319215000
H	-4.710898000	-1.241503000	3.933490000
C	-3.778925000	0.294719000	2.752381000
C	-1.743164000	-1.589734000	2.309568000
C	2.260160000	-2.608901000	-1.355217000
C	-2.843616000	-1.928154000	3.118516000
C	2.176776000	-1.239928000	-1.663250000
C	-3.383091000	-0.534116000	-1.306449000
C	1.345854000	1.537572000	1.112133000
C	-2.460544000	0.416091000	-1.785134000
C	1.813079000	0.297769000	1.424105000
C	4.543901000	-0.974790000	-1.476886000
H	5.439911000	-0.353544000	-1.506533000
C	3.294352000	-0.393024000	-1.706993000
C	3.164384000	1.091639000	-2.011402000

H	2.156056000	1.401330000	-1.694282000
C	-2.982690000	-1.978279000	-1.028870000
H	-1.995481000	-1.945964000	-0.542858000
C	3.534023000	-3.136941000	-1.132476000
H	3.645188000	-4.194928000	-0.891142000
C	-4.693547000	-0.097268000	-1.102203000
H	-5.438839000	-0.791279000	-0.714635000
C	-2.798861000	1.755465000	-2.036827000
C	1.027728000	-3.501036000	-1.287698000
H	0.168161000	-2.861775000	-1.031848000
C	4.664988000	-2.331847000	-1.203713000
H	5.651785000	-2.764418000	-1.026707000
C	-2.971255000	-3.301270000	3.724997000
H	-2.994033000	-4.085100000	2.949500000
H	-3.891561000	-3.391914000	4.319078000
H	-2.121010000	-3.536227000	4.385419000
C	-4.894949000	1.282097000	2.974930000
H	-5.698924000	0.847445000	3.585661000
H	-5.335159000	1.610106000	2.018684000
H	-4.538524000	2.191199000	3.485908000
C	-0.714232000	-2.656994000	2.030157000
H	-0.311806000	-3.100926000	2.955048000
H	0.144465000	-2.261625000	1.467806000
H	-1.149585000	-3.487987000	1.447701000

C	1.131980000	-4.568703000	-0.195761000
H	0.156345000	-5.057585000	-0.055471000
H	1.439980000	-4.136085000	0.766269000
H	1.852310000	-5.357373000	-0.463472000
C	-3.927419000	-2.683045000	-0.056975000
H	-3.481177000	-3.635756000	0.266100000
H	-4.893764000	-2.921918000	-0.529882000
H	-4.110293000	-2.078211000	0.840588000
C	-2.580251000	2.037299000	1.423775000
H	-1.652746000	2.201884000	0.855738000
H	-2.603597000	2.787322000	2.231640000
H	-3.431038000	2.255429000	0.760274000
C	4.155545000	1.931194000	-1.206825000
H	4.074127000	1.704773000	-0.136185000
H	3.946857000	3.001336000	-1.347774000
H	5.196592000	1.763085000	-1.523859000
C	-2.853704000	-2.800429000	-2.319230000
H	-2.583320000	-3.839537000	-2.075979000
H	-2.081659000	-2.411557000	-2.996388000
H	-3.810477000	-2.819790000	-2.864825000
C	-1.840988000	2.991033000	-4.019246000
H	-1.666103000	2.059163000	-4.574539000
H	-1.082199000	3.720458000	-4.341012000
H	-2.829674000	3.376626000	-4.314872000

C	0.561224000	3.891158000	2.814399000
H	-0.481388000	3.785513000	2.479626000
H	0.734064000	4.947877000	3.075556000
H	0.679908000	3.287312000	3.728106000
C	4.927996000	0.191428000	2.059090000
H	5.648690000	-0.223219000	2.783207000
H	5.035972000	1.285837000	2.065905000
H	5.206927000	-0.175867000	1.059979000
C	3.509315000	3.668061000	2.104386000
H	3.733937000	3.086365000	3.012167000
H	3.601806000	4.736082000	2.362278000
H	4.281732000	3.438325000	1.355066000
C	-4.133420000	2.127117000	-1.848210000
H	-4.439384000	3.155936000	-2.044832000
C	3.310886000	1.371445000	-3.512645000
H	4.304773000	1.060384000	-3.872154000
H	3.199274000	2.448027000	-3.713684000
H	2.555581000	0.841544000	-4.110043000
C	3.151255000	-2.239795000	2.484286000
H	3.936874000	-2.666556000	3.128628000
H	3.311216000	-2.605800000	1.458617000
H	2.178262000	-2.619798000	2.832861000
C	-5.069960000	1.211428000	-1.387886000
H	-6.105533000	1.522598000	-1.234633000

C	-1.774229000	2.781231000	-2.500906000
H	-0.774205000	2.392125000	-2.251748000
C	0.738292000	-4.153382000	-2.646338000
H	1.583529000	-4.787171000	-2.958212000
H	0.566391000	-3.406941000	-3.434323000
H	-0.159058000	-4.788006000	-2.586181000
C	-1.928018000	4.120446000	-1.773817000
H	-1.929290000	3.988928000	-0.682700000
H	-2.858390000	4.636203000	-2.057354000
H	-1.094703000	4.788287000	-2.036328000
C	1.453451000	4.345038000	-0.073585000
H	2.107073000	4.044485000	-0.906749000
H	1.616318000	5.418084000	0.116922000
H	0.412794000	4.210021000	-0.401113000
C	2.798708000	0.218813000	4.275482000
H	1.788475000	-0.106242000	4.571656000
H	2.824548000	1.318068000	4.344251000
H	3.519239000	-0.187626000	5.003738000

Compound 6, ωB97X-D/Def2-SVP

Geometry of the ground state

Energy = –3579.58442790E$_h$

C	-2.707629000	-4.033354000	-0.229661000
H	-2.542544000	-5.054808000	-0.556217000

C	-3.760023000	-3.446643000	0.377414000
H	-4.720348000	-3.840778000	0.694172000
N	-3.429568000	-2.117048000	0.551917000
N	-1.748922000	-3.053850000	-0.409459000
C	-2.176796000	-1.840032000	0.061594000
Al	-1.212488000	-0.005920000	-0.014346000
Al	1.177055000	0.065074000	0.059765000
C	2.118700000	1.881116000	0.321555000
N	1.635720000	3.133355000	0.034270000
N	3.324338000	2.135967000	0.934892000
C	2.494390000	4.118116000	0.487669000
H	2.275426000	5.171314000	0.347517000
C	3.544450000	3.494184000	1.060556000
H	4.437265000	3.886230000	1.536313000
C	-0.508079000	-3.418340000	-1.046490000
C	1.636475000	-4.529273000	-2.410002000
C	0.439184000	-4.167550000	-0.325870000
C	-0.387060000	-3.195813000	-2.434116000
C	0.697231000	-3.768975000	-3.096767000
C	1.514278000	-4.704437000	-1.043464000
H	0.804314000	-3.628494000	-4.173462000
H	2.276780000	-5.268702000	-0.501983000
H	2.476424000	-4.978920000	-2.942423000
C	-4.347493000	-1.249863000	1.245029000

C	-6.225462000	0.194781000	2.690954000
C	-4.209909000	-1.146300000	2.647859000
C	-5.419276000	-0.659575000	0.554596000
C	-6.321637000	0.101125000	1.312042000
C	-5.183616000	-0.443370000	3.355041000
H	-7.134001000	0.616022000	0.793453000
H	-5.106699000	-0.364143000	4.440617000
H	-6.963159000	0.771938000	3.252795000
C	0.490497000	3.506973000	-0.757727000
C	-1.512711000	4.552584000	-2.376454000
C	-0.581011000	4.192074000	-0.159061000
C	0.574289000	3.334872000	-2.159819000
C	-0.438133000	3.877905000	-2.946842000
C	-1.581477000	4.691891000	-1.003687000
H	-0.390949000	3.771153000	-4.031055000
H	-2.444768000	5.188797000	-0.555186000
H	-2.306530000	4.957381000	-3.007392000
C	4.360617000	1.189565000	1.262909000
C	6.403129000	-0.613194000	1.799682000
C	5.250283000	0.821510000	0.225164000
C	4.531043000	0.735661000	2.579951000
C	5.571188000	-0.176281000	2.814670000
C	6.250816000	-0.102511000	0.515436000
H	5.711615000	-0.556263000	3.830369000

H	6.926917000	-0.433514000	-0.272428000
H	7.184266000	-1.347338000	2.007457000
C	-0.774892000	4.511906000	1.321092000
H	-1.857545000	4.378077000	1.470722000
C	-0.475908000	5.991853000	1.604977000
H	0.594273000	6.217093000	1.474376000
H	-0.740434000	6.241807000	2.644264000
H	-1.042553000	6.658368000	0.938568000
C	-0.074243000	3.621887000	2.342237000
H	0.973846000	3.918215000	2.474016000
H	-0.103647000	2.556836000	2.067710000
H	-0.560851000	3.732786000	3.323490000
C	1.763287000	2.655125000	-2.823094000
H	2.119224000	1.876441000	-2.134841000
C	1.394838000	1.937012000	-4.120569000
H	0.536911000	1.266200000	-3.968093000
H	2.243962000	1.329527000	-4.466704000
H	1.147246000	2.638849000	-4.932439000
C	2.909907000	3.647309000	-3.051079000
H	2.595593000	4.460760000	-3.724396000
H	3.772338000	3.138799000	-3.510759000
H	3.252704000	4.099482000	-2.108706000
C	3.769918000	1.192589000	3.822477000
H	3.801157000	0.324238000	4.501348000

C	5.197558000	1.472156000	-1.151362000
H	4.140657000	1.651794000	-1.398050000
C	5.931819000	2.822654000	-1.146375000
H	5.917226000	3.266790000	-2.153390000
H	6.984116000	2.680075000	-0.853254000
H	5.482680000	3.549779000	-0.459859000
C	5.792138000	0.601729000	-2.257443000
H	5.520677000	1.008799000	-3.243443000
H	5.429437000	-0.429114000	-2.190590000
H	6.892419000	0.581863000	-2.209476000
C	2.302397000	1.577574000	3.671683000
H	2.196433000	2.578893000	3.241059000
H	1.826393000	1.600225000	4.663865000
H	1.734094000	0.881052000	3.038902000
C	4.548839000	2.322113000	4.514487000
H	4.560445000	3.228485000	3.888761000
H	5.592191000	2.035669000	4.713078000
H	4.074547000	2.586043000	5.472431000
C	0.400658000	-4.535714000	1.155489000
H	1.458173000	-4.487398000	1.461769000
C	-1.447071000	-2.444044000	-3.221803000
H	-1.949180000	-1.759550000	-2.526173000
C	-0.050855000	-5.991956000	1.342225000
H	-1.108574000	-6.116927000	1.062120000

H	0.048899000	-6.291340000	2.397219000
H	0.545134000	-6.686018000	0.731823000
C	-0.371841000	-3.618537000	2.097626000
H	-1.451980000	-3.807225000	2.043923000
H	-0.188740000	-2.554425000	1.889289000
H	-0.065676000	-3.817445000	3.136596000
C	-2.501096000	-3.409986000	-3.776401000
H	-2.046879000	-4.119583000	-4.486629000
H	-3.292856000	-2.856081000	-4.304335000
H	-2.978099000	-3.993738000	-2.975216000
C	-0.853607000	-1.581463000	-4.335091000
H	-1.636814000	-0.954197000	-4.785289000
H	-0.418091000	-2.187270000	-5.145112000
H	-0.070582000	-0.919074000	-3.939883000
C	-3.062927000	-1.811668000	3.391572000
H	-2.233553000	-1.911946000	2.681141000
C	-2.524417000	-0.984354000	4.561003000
H	-1.607950000	-1.453375000	4.949656000
H	-3.239482000	-0.926459000	5.397126000
H	-2.270682000	0.037936000	4.249892000
C	-3.466069000	-3.212619000	3.869382000
H	-2.623126000	-3.704819000	4.378189000
H	-3.774449000	-3.857862000	3.034277000
H	-4.306968000	-3.153690000	4.579111000

C	-5.779625000	-0.802759000	-0.925271000
H	-6.022758000	0.225085000	-1.243607000
C	-4.725295000	-1.315875000	-1.901194000
H	-4.570034000	-2.401156000	-1.822266000
H	-3.766458000	-0.812914000	-1.758549000
H	-5.062094000	-1.111608000	-2.929202000
C	-7.052268000	-1.654758000	-1.061597000
H	-7.887296000	-1.261070000	-0.464840000
H	-6.861584000	-2.688338000	-0.729489000
H	-7.374375000	-1.695625000	-2.113528000
C	-2.664136000	1.383036000	-0.309840000
C	-4.805398000	3.149739000	-0.793370000
C	-3.094420000	1.667133000	-1.630507000
C	-3.305048000	2.062038000	0.752822000
C	-4.364602000	2.958144000	0.514395000
C	-4.187774000	2.520709000	-1.872180000
H	-5.648423000	3.824051000	-0.979371000
C	-2.398767000	1.083863000	-2.835769000
H	-1.495089000	0.529267000	-2.542138000
H	-2.082331000	1.876445000	-3.530383000
H	-3.052195000	0.406578000	-3.409605000
C	-4.684670000	2.765850000	-3.273385000
H	-4.972363000	1.825578000	-3.772095000
H	-3.908302000	3.226360000	-3.906687000

H	-5.560306000	3.430881000	-3.277715000
C	-5.030454000	3.691275000	1.649861000
H	-5.454904000	2.991410000	2.387730000
H	-5.843541000	4.337098000	1.288128000
H	-4.315402000	4.327751000	2.198073000
C	-2.893797000	1.851806000	2.186438000
H	-2.058800000	1.138930000	2.264250000
H	-3.729392000	1.472639000	2.787162000
H	-2.576810000	2.791041000	2.663381000
C	2.600263000	-1.365758000	-0.101926000
C	4.631579000	-3.283427000	-0.434559000
C	3.084040000	-2.109175000	0.999967000
C	3.137544000	-1.645026000	-1.383331000
C	4.158219000	-2.600856000	-1.553413000
C	4.104781000	-3.065809000	0.836555000
H	5.428317000	-4.024570000	-0.560191000
C	2.625666000	-0.934699000	-2.612493000
H	3.330200000	-0.163614000	-2.965892000
H	1.663264000	-0.440519000	-2.408378000
H	2.466579000	-1.628846000	-3.451111000
C	4.719563000	-2.922297000	-2.916621000
H	3.946114000	-3.338548000	-3.582290000
H	5.533710000	-3.658271000	-2.847921000
H	5.114957000	-2.032530000	-3.431168000

C	2.530529000	-1.918245000	2.390179000
H	2.164777000	-2.865490000	2.815715000
H	1.691740000	-1.206833000	2.394785000
H	3.299911000	-1.548970000	3.084230000
C	4.627136000	-3.848869000	2.012805000
H	5.003944000	-3.181528000	2.805006000
H	5.446995000	-4.518968000	1.716385000
H	3.838995000	-4.467632000	2.474603000

Compound 7, ωB97X-D/Def2-SVP

Geometry of the ground state

Energy = –2420.62064667E_h

C	-0.202339000	2.026213000	2.492930000
H	0.414816000	2.477897000	3.262801000
C	-1.527782000	2.121024000	2.246237000
H	-2.311189000	2.667893000	2.761217000
N	-1.791818000	1.329867000	1.142976000
N	0.330651000	1.179895000	1.533887000
C	-0.650291000	0.715154000	0.688014000
Al	-0.484482000	-0.586411000	-0.812273000
Al	1.833768000	-0.728901000	-1.148087000
C	1.718958000	0.820194000	1.507311000
C	4.418709000	0.174578000	1.715477000
C	2.675513000	1.715169000	0.984196000

C	2.099584000	-0.391485000	2.141842000
C	3.458366000	-0.685771000	2.241235000
C	4.028398000	1.351081000	1.101160000
H	3.772692000	-1.605455000	2.736664000
H	4.787301000	2.019251000	0.687142000
H	5.477958000	-0.082703000	1.774352000
C	-3.080248000	1.223926000	0.514638000
C	-5.516257000	1.019948000	-0.793078000
C	-3.355759000	2.091818000	-0.566813000
C	-4.016663000	0.283855000	0.976912000
C	-5.228853000	0.193746000	0.280891000
C	-4.591065000	1.974039000	-1.202249000
H	-5.960178000	-0.553908000	0.597240000
H	-4.830873000	2.628903000	-2.041145000
H	-6.469011000	0.920573000	-1.317349000
C	2.414555000	3.110721000	0.420435000
H	3.233109000	3.267376000	-0.299492000
C	1.068472000	-1.286394000	2.807923000
H	0.115056000	-1.110745000	2.298548000
C	2.594938000	4.165732000	1.522540000
H	1.801168000	4.084494000	2.280796000
H	2.540385000	5.178493000	1.094401000
H	3.563977000	4.059080000	2.032073000
C	1.114075000	3.345122000	-0.343637000

H	0.265207000	3.494341000	0.338291000
H	0.876485000	2.504988000	-1.015064000
H	1.205735000	4.259954000	-0.949171000
C	0.897236000	-0.899705000	4.282427000
H	1.832011000	-1.056186000	4.844231000
H	0.111070000	-1.512542000	4.749889000
H	0.608938000	0.156136000	4.394132000
C	1.367857000	-2.776136000	2.656382000
H	0.525172000	-3.366223000	3.047421000
H	2.268239000	-3.085447000	3.210762000
H	1.500107000	-3.035073000	1.596152000
C	-2.362989000	3.166190000	-0.986868000
H	-1.360569000	2.745780000	-0.837312000
C	-2.451706000	3.577555000	-2.456709000
H	-1.616560000	4.252271000	-2.697633000
H	-3.381987000	4.124311000	-2.679294000
H	-2.388140000	2.713705000	-3.129656000
C	-2.502144000	4.411787000	-0.099818000
H	-1.753995000	5.168912000	-0.381673000
H	-2.358289000	4.184054000	0.964523000
H	-3.502015000	4.859272000	-0.217921000
C	-3.907788000	-0.590295000	2.224995000
H	-4.433460000	-1.520788000	1.953220000
C	-2.522661000	-1.008707000	2.711955000

H	-1.996056000	-0.198648000	3.236501000
H	-1.892922000	-1.351759000	1.885544000
H	-2.626251000	-1.841488000	3.424619000
C	-4.692530000	0.065234000	3.371933000
H	-5.731664000	0.282624000	3.085239000
H	-4.220800000	1.015705000	3.669537000
H	-4.708801000	-0.592118000	4.255121000
C	-2.150999000	-1.568889000	-1.321247000
C	-4.666502000	-2.727255000	-1.768282000
C	-2.566946000	-2.701732000	-0.577738000
C	-2.990606000	-1.097330000	-2.356877000
C	-4.253758000	-1.675269000	-2.582377000
C	-3.841470000	-3.264680000	-0.782380000
H	-5.657021000	-3.165951000	-1.928768000
C	-1.648970000	-3.397137000	0.400528000
H	-0.720847000	-2.829323000	0.561122000
H	-1.355654000	-4.386497000	0.009070000
H	-2.123990000	-3.573794000	1.377887000
C	-4.311231000	-4.445936000	0.028567000
H	-4.327536000	-4.220064000	1.107867000
H	-3.648499000	-5.317156000	-0.098864000
H	-5.325700000	-4.751782000	-0.264334000
C	-5.154404000	-1.174306000	-3.681113000
H	-5.406359000	-0.109813000	-3.542614000

H	-6.095691000	-1.741290000	-3.715429000
H	-4.674830000	-1.258445000	-4.669698000
C	-2.547088000	0.000680000	-3.288668000
H	-1.624836000	0.487869000	-2.934893000
H	-3.320955000	0.772797000	-3.409934000
H	-2.335630000	-0.402799000	-4.293859000
C	3.784006000	-0.847509000	-1.422379000
C	6.576125000	-0.870324000	-1.477043000
C	4.496402000	0.233269000	-1.993339000
C	4.499637000	-1.954598000	-0.911054000
C	5.906465000	-1.963864000	-0.930479000
C	5.902435000	0.223723000	-2.019749000
H	7.671015000	-0.875268000	-1.490861000
C	3.792443000	-3.152269000	-0.326894000
H	4.025695000	-4.069249000	-0.892039000
H	2.698090000	-3.028616000	-0.335598000
H	4.094326000	-3.334631000	0.717122000
C	6.684512000	-3.125961000	-0.369421000
H	6.462524000	-3.284789000	0.698587000
H	7.767458000	-2.966006000	-0.466576000
H	6.436141000	-4.067435000	-0.885130000
C	3.786250000	1.426449000	-2.585058000
H	4.139969000	2.371646000	-2.142592000
H	2.695337000	1.376656000	-2.440903000

H	3.969597000	1.500598000	-3.669753000
C	6.679084000	1.370241000	-2.614389000
H	6.411972000	1.537210000	-3.670125000
H	7.761518000	1.186199000	-2.567396000
H	6.476853000	2.316327000	-2.085574000

Compound 8, ωB97X-D/Def2-SVP

Geometry of the ground state

Energy = –4210.46960317E$_h$

Al	-2.505359000	0.228711000	-0.292108000
Al	2.306983000	0.062708000	0.255362000
Al	-0.187262000	-0.160097000	0.198767000
N	4.960947000	1.062767000	-1.011791000
N	3.669295000	0.317063000	-2.550414000
N	-4.990990000	1.272426000	1.096474000
N	-3.664256000	2.909460000	0.625853000
C	5.645594000	1.204965000	-2.201258000
H	6.629049000	1.660922000	-2.248145000
C	4.833193000	0.723486000	-3.171426000
H	4.960875000	0.650646000	-4.246685000
C	2.555696000	-1.408832000	-3.876813000
C	-3.430774000	-1.437311000	-0.975649000
C	3.340510000	-0.887221000	1.668151000
C	-3.812929000	-1.452149000	-2.340080000

C	3.737095000	0.519105000	-1.214282000
C	1.511454000	-1.748348000	-4.741570000
H	1.498585000	-2.735956000	-5.206796000
C	0.470938000	-0.862930000	-4.986899000
H	-0.350393000	-1.158527000	-5.642628000
C	2.527672000	-0.125025000	-3.312832000
C	3.517182000	-0.330786000	2.950010000
C	6.722370000	1.498074000	2.222885000
H	7.374087000	0.948826000	2.903212000
C	0.451199000	0.388072000	-4.378268000
H	-0.390125000	1.057558000	-4.556690000
C	5.424116000	1.633195000	0.224331000
C	-3.758499000	1.542893000	0.539544000
C	-4.800207000	3.462466000	1.194726000
H	-4.905496000	4.534435000	1.329542000
C	-5.627551000	2.438269000	1.490701000
H	-6.597674000	2.420850000	1.977546000
C	-3.932773000	-0.171124000	-3.132533000
H	-3.211140000	-0.130132000	-3.966873000
H	-3.745616000	0.711670000	-2.503082000
H	-4.939289000	-0.062703000	-3.572856000
C	6.339952000	2.797580000	2.543429000
H	6.699247000	3.254367000	3.468105000
C	1.489494000	0.798304000	-3.541065000

C	5.500609000	3.514857000	1.700032000
H	5.206989000	4.532315000	1.966864000
C	3.667505000	-2.406552000	-3.587844000
H	4.259143000	-2.018532000	-2.744284000
C	6.275744000	0.882391000	1.051878000
C	3.610034000	-2.255343000	1.471102000
C	4.154870000	-2.494441000	3.803534000
H	4.460011000	-3.126494000	4.643846000
C	-2.588669000	3.734959000	0.178316000
C	6.718607000	-0.522476000	0.674662000
H	5.888201000	-0.979608000	0.114011000
C	0.124638000	-2.184579000	0.423051000
C	3.117571000	-3.770632000	-3.157103000
H	2.410281000	-3.677397000	-2.321582000
H	3.940766000	-4.425915000	-2.834499000
H	2.598814000	-4.278610000	-3.985023000
C	5.027226000	2.949408000	0.512674000
C	-7.016873000	-1.672225000	-1.375736000
H	-7.980885000	-2.123765000	-1.088897000
H	-6.233568000	-2.438901000	-1.306658000
H	-7.100306000	-1.371756000	-2.431340000
C	6.988528000	-1.409908000	1.888739000
H	7.928614000	-1.132779000	2.392770000
H	7.088045000	-2.458035000	1.570007000

H	6.169564000	-1.357587000	2.616129000
C	-4.041125000	-3.850941000	-2.287421000
H	-4.262926000	-4.793493000	-2.799619000
C	7.957780000	-0.487055000	-0.231778000
H	7.781494000	0.054618000	-1.170558000
H	8.266322000	-1.510831000	-0.492625000
H	8.800292000	-0.001086000	0.285332000
C	-4.103435000	-2.656933000	-3.005493000
C	4.609098000	-2.549760000	-4.789738000
H	5.057031000	-1.586829000	-5.076988000
H	4.068138000	-2.938588000	-5.666769000
H	5.427607000	-3.249123000	-4.560476000
C	3.938526000	-1.132583000	4.026198000
C	-0.031695000	-3.078655000	-0.663236000
C	4.004084000	-3.074425000	2.543729000
C	-2.720566000	4.378579000	-1.061764000
C	-1.509519000	3.966394000	1.049643000
C	-3.871419000	4.064740000	-2.004605000
H	-4.430870000	3.220594000	-1.575936000
C	0.415647000	-1.848232000	2.934944000
H	-0.423395000	-2.069283000	3.616780000
H	0.348019000	-0.780232000	2.678110000
H	1.347165000	-2.001145000	3.500563000
C	1.514126000	2.197275000	-2.941661000

H	1.939671000	2.111733000	-1.931102000
C	-3.418498000	-2.665641000	-0.275035000
C	0.347768000	-2.727212000	1.710593000
C	-5.405489000	0.008642000	1.637963000
C	-4.077791000	0.620775000	3.749785000
H	-3.825024000	1.486584000	3.124176000
C	-1.768200000	5.347002000	-1.392365000
H	-1.849325000	5.881007000	-2.342177000
C	-4.994994000	-0.293350000	2.948663000
C	-6.257638000	-0.825333000	0.896352000
C	-3.074193000	-2.738201000	1.188626000
H	-3.937831000	-3.085362000	1.777797000
H	-2.769930000	-1.756145000	1.574602000
H	-2.244426000	-3.436389000	1.374235000
C	-0.577775000	4.934377000	0.667687000
H	0.264732000	5.157470000	1.323649000
C	4.127360000	3.751201000	-0.416935000
H	4.001857000	3.183321000	-1.349414000
C	-6.686191000	-0.454169000	-0.513917000
H	-5.827192000	0.053212000	-0.978122000
C	-0.716617000	5.632437000	-0.529376000
H	0.014610000	6.398884000	-0.798063000
C	-3.369026000	3.607071000	-3.376544000
H	-4.217528000	3.321149000	-4.016911000

H	-2.709317000	2.733956000	-3.273869000
H	-2.810986000	4.403285000	-3.895232000
C	-7.867473000	0.525658000	-0.497803000
H	-8.165921000	0.780950000	-1.526622000
H	-7.621814000	1.464140000	0.018526000
H	-8.738743000	0.077138000	0.006204000
C	3.201128000	1.120399000	3.211099000
H	2.967240000	1.667471000	2.285139000
H	4.042788000	1.646418000	3.686696000
H	2.329082000	1.227065000	3.877177000
C	2.411875000	3.138911000	-3.757476000
H	2.445997000	4.132552000	-3.283296000
H	2.012488000	3.265311000	-4.776210000
H	3.445558000	2.775130000	-3.843956000
C	-6.711890000	-1.992411000	1.515373000
H	-7.366075000	-2.672359000	0.968579000
C	-1.405356000	3.230694000	2.378623000
H	-1.858291000	2.237390000	2.228543000
C	3.446770000	-2.879622000	0.110044000
H	2.718190000	-3.705068000	0.127022000
H	4.400365000	-3.281275000	-0.275111000
H	3.074192000	-2.160226000	-0.637334000
C	4.121912000	-0.547862000	5.402352000
H	3.188272000	-0.098797000	5.778221000

H	4.880529000	0.252271000	5.404057000
H	4.439772000	-1.313519000	6.123915000
C	-3.724073000	-3.875664000	-0.929671000
C	0.023759000	-4.471919000	-0.468690000
C	-6.337003000	-2.307238000	2.818004000
H	-6.706189000	-3.224759000	3.281672000
C	-4.835615000	5.250064000	-2.119620000
H	-5.690684000	4.992783000	-2.763389000
H	-4.337718000	6.130346000	-2.557007000
H	-5.229168000	5.542668000	-1.134534000
C	0.035511000	2.970927000	2.809521000
H	0.584101000	2.427118000	2.025285000
H	0.041210000	2.337162000	3.709438000
H	0.581122000	3.896606000	3.056368000
C	-0.322848000	-2.573592000	-2.051301000
H	0.260391000	-3.107810000	-2.814675000
H	-0.108907000	-1.498366000	-2.146970000
H	-1.385477000	-2.711465000	-2.306858000
C	0.453389000	-4.117097000	1.905335000
C	-4.487896000	-2.671320000	-4.462626000
H	-4.631051000	-3.698699000	-4.827311000
H	-3.719464000	-2.194585000	-5.093245000
H	-5.426441000	-2.119886000	-4.641530000
C	-5.483208000	-1.469008000	3.524429000

H	-5.183554000	-1.733942000	4.541226000
C	4.243244000	-4.546262000	2.339284000
H	3.342509000	-5.038930000	1.938129000
H	4.513066000	-5.044487000	3.281128000
H	5.055208000	-4.728700000	1.615945000
C	-2.184062000	3.957275000	3.482466000
H	-3.248843000	4.068204000	3.231172000
H	-1.769462000	4.962508000	3.662687000
H	-2.124274000	3.390651000	4.424907000
C	0.130693000	2.810077000	-2.766503000
H	0.209973000	3.731010000	-2.174173000
H	-0.547770000	2.126209000	-2.229584000
H	-0.322736000	3.084353000	-3.732652000
C	0.287207000	-4.965828000	0.810220000
H	0.340420000	-6.049380000	0.962735000
C	-2.751647000	-0.060577000	4.096632000
H	-2.902215000	-0.972013000	4.697316000
H	-2.113851000	0.624011000	4.678797000
H	-2.204108000	-0.327613000	3.180129000
C	-4.788020000	1.163299000	4.994261000
H	-5.723309000	1.678880000	4.726935000
H	-4.141847000	1.882275000	5.521517000
H	-5.039029000	0.357885000	5.702614000
C	-3.684048000	-5.184949000	-0.185732000

H	-3.952375000	-6.027045000	-0.840317000
H	-4.378888000	-5.183826000	0.669835000
H	-2.678797000	-5.375766000	0.224968000
C	-0.235179000	-5.424414000	-1.608322000
H	-0.218234000	-6.469583000	-1.266562000
H	0.512974000	-5.322087000	-2.412132000
H	-1.219458000	-5.229757000	-2.065193000
C	2.729628000	3.946144000	0.175749000
H	2.768887000	4.503171000	1.125535000
H	2.224657000	2.986998000	0.374652000
H	2.088375000	4.504247000	-0.522538000
C	0.694301000	-4.689672000	3.278475000
H	0.786538000	-5.785128000	3.244869000
H	-0.130436000	-4.443445000	3.967694000
H	1.613301000	-4.282325000	3.730648000
C	4.761837000	5.090170000	-0.805837000
H	4.139191000	5.599774000	-1.556811000
H	5.765853000	4.948954000	-1.233296000
H	4.855220000	5.765829000	0.058465000

Compound 9, ωB97X-D/Def2-SVP

Geometry of the ground state

Energy = –4210.50840426E$_h$

Al	-2.380560000	0.020452000	-0.472406000

Al	2.518433000	0.556039000	0.394764000
Al	0.168220000	0.108999000	-0.024953000
N	2.047621000	-0.399720000	-2.370209000
N	4.183335000	-0.532961000	-2.103480000
N	-4.572041000	0.711956000	1.648542000
N	-3.944174000	2.491462000	0.636199000
C	2.593153000	-0.678776000	-3.602435000
H	1.984035000	-0.774669000	-4.492970000
C	3.935289000	-0.774028000	-3.440113000
H	4.733856000	-0.983804000	-4.144855000
C	6.167976000	0.610758000	-1.217671000
C	-3.369109000	-1.753339000	-0.706958000
C	4.078744000	1.047687000	1.546888000
C	-4.363029000	-1.857343000	-1.712410000
C	3.023388000	-0.308537000	-1.434147000
C	7.447064000	0.496248000	-0.668822000
H	7.992186000	1.392823000	-0.376729000
C	8.048963000	-0.745544000	-0.486514000
H	9.053829000	-0.802409000	-0.062441000
C	5.500542000	-0.588636000	-1.537270000
C	4.248854000	2.410400000	1.886865000
C	-0.931290000	1.792381000	-2.425299000
H	-1.185440000	2.848230000	-2.549290000
C	7.380352000	-1.907527000	-0.844841000

H	7.864623000	-2.876178000	-0.702493000
C	0.630913000	-0.075301000	-2.118006000
C	-3.732985000	1.151925000	0.672174000
C	-4.882423000	2.883238000	1.570529000
H	-5.172682000	3.921240000	1.694187000
C	-5.272915000	1.760832000	2.212666000
H	-5.961507000	1.608985000	3.036406000
C	-4.944010000	-0.632701000	-2.379596000
H	-4.575701000	-0.498880000	-3.410817000
H	-4.694574000	0.279354000	-1.827639000
H	-6.041403000	-0.690241000	-2.443837000
C	-2.043321000	0.803091000	-2.364867000
H	-2.949398000	1.211284000	-2.828123000
C	6.084167000	-1.854225000	-1.365797000
C	-1.608137000	-0.462850000	-3.023407000
H	-2.361463000	-1.068570000	-3.533325000
C	5.546323000	1.977233000	-1.489149000
H	4.513483000	1.949037000	-1.107196000
C	0.358666000	1.408731000	-2.394114000
C	4.987180000	0.103096000	2.071322000
C	6.174929000	1.855580000	3.226550000
H	6.995318000	2.169941000	3.880296000
C	-3.281393000	3.445729000	-0.209003000
C	1.494426000	2.395413000	-2.625340000

H	2.411247000	1.983870000	-2.174874000
C	1.188973000	-1.251959000	1.312161000
C	6.255104000	3.119527000	-0.763962000
H	6.342639000	2.924538000	0.313763000
H	5.683307000	4.049813000	-0.897547000
H	7.261505000	3.298065000	-1.175403000
C	-0.353176000	-0.935648000	-2.909214000
C	-7.542360000	-1.680840000	-0.319222000
H	-8.294660000	-2.310341000	0.181267000
H	-6.776698000	-2.337069000	-0.751470000
H	-8.060775000	-1.154130000	-1.134917000
C	1.270426000	3.753973000	-1.964320000
H	0.457792000	4.310882000	-2.454592000
H	2.181546000	4.370776000	-2.030349000
H	1.002986000	3.631956000	-0.906669000
C	-4.414122000	-4.257084000	-1.483661000
H	-4.799802000	-5.231506000	-1.801341000
C	1.766208000	2.582970000	-4.125268000
H	1.961459000	1.628035000	-4.631684000
H	2.637365000	3.237648000	-4.292123000
H	0.890791000	3.041178000	-4.611832000
C	-4.849401000	-3.107344000	-2.140159000
C	5.477139000	2.283547000	-2.992466000
H	4.857804000	1.563924000	-3.543057000

H	6.485970000	2.282655000	-3.435072000
H	5.037012000	3.279800000	-3.151562000
C	5.294933000	2.818449000	2.736663000
C	1.563582000	-2.587653000	1.011857000
C	6.045095000	0.504815000	2.907042000
C	-3.862354000	3.765206000	-1.447626000
C	-2.137645000	4.086882000	0.294246000
C	-5.163173000	3.126681000	-1.908828000
H	-5.253171000	2.156421000	-1.400062000
C	0.887850000	0.574756000	3.080785000
H	0.656055000	0.627514000	4.152640000
H	-0.003839000	0.905866000	2.529088000
H	1.676076000	1.324196000	2.894887000
C	5.365756000	-3.142623000	-1.739383000
H	4.325255000	-2.891109000	-1.989161000
C	-2.958950000	-2.947149000	-0.071134000
C	1.315045000	-0.818902000	2.666302000
C	-4.794640000	-0.643221000	2.092293000
C	-2.658843000	-0.492248000	3.528135000
H	-2.119757000	-0.124570000	2.636529000
C	-3.240428000	4.757626000	-2.208326000
H	-3.653977000	5.031049000	-3.180418000
C	-3.909651000	-1.213569000	3.026861000
C	-5.943681000	-1.306160000	1.621181000

C	-1.893483000	-2.956857000	0.993183000
H	-2.230264000	-3.485025000	1.897331000
H	-1.579923000	-1.947354000	1.281428000
H	-0.986839000	-3.478911000	0.647112000
C	-1.564791000	5.082263000	-0.500925000
H	-0.674881000	5.606592000	-0.150049000
C	-0.027659000	-2.314815000	-3.488884000
H	1.010444000	-2.575823000	-3.229344000
C	-6.926023000	-0.666369000	0.647884000
H	-6.367307000	0.068625000	0.046997000
C	-2.105728000	5.410473000	-1.738379000
H	-1.635530000	6.186392000	-2.346319000
C	-5.213653000	2.856933000	-3.413853000
H	-6.096587000	2.246295000	-3.654736000
H	-4.322582000	2.315498000	-3.760526000
H	-5.294234000	3.789578000	-3.993843000
C	-8.058086000	0.066526000	1.384013000
H	-8.762276000	0.503934000	0.659439000
H	-7.697334000	0.879609000	2.025451000
H	-8.619440000	-0.638247000	2.017675000
C	3.345990000	3.481993000	1.324528000
H	2.561938000	3.063317000	0.676947000
H	3.915129000	4.213567000	0.727652000
H	2.840069000	4.050513000	2.121615000

C	5.996444000	-3.775340000	-2.985519000
H	5.438466000	-4.674510000	-3.288413000
H	7.039595000	-4.073168000	-2.793932000
H	6.001827000	-3.076394000	-3.834984000
C	-6.182834000	-2.597047000	2.095383000
H	-7.049228000	-3.152236000	1.735226000
C	-1.600706000	3.791760000	1.687947000
H	-1.962048000	2.794716000	1.983890000
C	4.858133000	-1.363162000	1.761153000
H	4.573423000	-1.937884000	2.656548000
H	5.807089000	-1.778542000	1.393114000
H	4.091285000	-1.560920000	1.000519000
C	5.487650000	4.270631000	3.089351000
H	4.594477000	4.695519000	3.575070000
H	5.677626000	4.882432000	2.191536000
H	6.337903000	4.406160000	3.772586000
C	-3.497259000	-4.196495000	-0.438596000
C	1.982962000	-3.475161000	2.016439000
C	-5.327673000	-3.189439000	3.013280000
H	-5.528706000	-4.201334000	3.371333000
C	-6.362428000	3.983638000	-1.479240000
H	-7.306668000	3.505800000	-1.782690000
H	-6.316817000	4.978812000	-1.949357000
H	-6.394133000	4.127900000	-0.390073000

C	-0.076847000	3.741605000	1.747464000
H	0.319410000	2.963151000	1.076955000
H	0.250393000	3.498046000	2.769521000
H	0.382098000	4.707072000	1.483003000
C	1.499235000	-3.095264000	-0.401941000
H	2.508972000	-3.220962000	-0.828346000
H	0.952857000	-2.393958000	-1.036687000
H	1.002252000	-4.074754000	-0.473965000
C	1.840929000	-1.672338000	3.655836000
C	-5.821993000	-3.220122000	-3.287570000
H	-6.022755000	-4.272282000	-3.534595000
H	-5.434907000	-2.730070000	-4.195341000
H	-6.789914000	-2.741737000	-3.061186000
C	-4.213408000	-2.502416000	3.476140000
H	-3.554926000	-2.992862000	4.191138000
C	7.030355000	-0.499250000	3.446398000
H	6.528424000	-1.295749000	4.018758000
H	7.767386000	-0.021655000	4.107536000
H	7.580338000	-0.995390000	2.629337000
C	-2.149806000	4.812812000	2.694393000
H	-3.248899000	4.838568000	2.702601000
H	-1.793752000	5.826768000	2.451802000
H	-1.810952000	4.568333000	3.712777000
C	5.313308000	-4.141180000	-0.579932000

H	4.735990000	-5.030696000	-0.875852000
H	4.831950000	-3.701486000	0.304355000
H	6.317383000	-4.484273000	-0.285711000
C	2.131008000	-2.991674000	3.315352000
H	2.505991000	-3.667105000	4.091354000
C	-1.703399000	-1.414970000	4.284861000
H	-2.154960000	-1.786318000	5.218589000
H	-0.803600000	-0.856400000	4.564438000
H	-1.377435000	-2.272554000	3.681763000
C	-2.976594000	0.720397000	4.415954000
H	-3.541231000	1.502821000	3.894404000
H	-2.038455000	1.177443000	4.766322000
H	-3.554430000	0.412969000	5.302417000
C	-3.057169000	-5.462619000	0.249235000
H	-3.554079000	-6.344339000	-0.179987000
H	-3.285781000	-5.437159000	1.327562000
H	-1.968403000	-5.613602000	0.162759000
C	2.273281000	-4.922795000	1.713800000
H	2.661909000	-5.445398000	2.599287000
H	3.009426000	-5.036198000	0.904323000
H	1.361185000	-5.450341000	1.388043000
C	-0.923028000	-3.414247000	-2.900338000
H	-1.979098000	-3.260924000	-3.170723000
H	-0.883233000	-3.424544000	-1.804808000

H	-0.618300000	-4.407144000	-3.269976000
C	2.114873000	-1.193920000	5.059979000
H	2.624618000	-1.972230000	5.645177000
H	1.192297000	-0.928282000	5.602283000
H	2.758738000	-0.300482000	5.056217000
C	-0.143275000	-2.336511000	-5.020129000
H	0.173137000	-3.309887000	-5.428957000
H	0.455586000	-1.550044000	-5.504606000
H	-1.187379000	-2.167046000	-5.324837000

Compound 9’, ωB97X-D/Def2-SVP

Geometry of the ground state

Energy = –4210.44201107E$_h$

C	-5.823406000	-0.965861000	1.331482000
H	-6.595699000	-1.724261000	1.264870000
C	-5.601874000	0.007952000	2.233138000
H	-6.151210000	0.311769000	3.117732000
N	-4.459261000	0.672270000	1.830115000
N	-4.817346000	-0.876300000	0.392975000
C	-3.932672000	0.133279000	0.685559000
Al	-1.994978000	0.287069000	-0.391010000
Al	0.133377000	-0.344302000	0.865383000
Al	2.334137000	-0.112887000	-0.155426000
C	3.742931000	-1.450345000	0.479235000

N	3.469286000	-2.612011000	1.155130000
N	5.089141000	-1.530112000	0.224860000
C	4.607721000	-3.380816000	1.312405000
H	4.589206000	-4.329797000	1.839056000
C	5.621361000	-2.703545000	0.728089000
H	6.675825000	-2.940499000	0.631200000
C	-4.831098000	-1.840099000	-0.686382000
C	-4.975953000	-3.701800000	-2.732053000
C	-4.318007000	-3.126017000	-0.460768000
C	-5.510327000	-1.496653000	-1.879256000
C	-5.548789000	-2.444823000	-2.897266000
C	-4.393380000	-4.037763000	-1.524581000
H	-6.026926000	-2.201617000	-3.845109000
H	-3.974256000	-5.037943000	-1.385926000
H	-5.001705000	-4.424839000	-3.549817000
C	-4.065067000	1.779108000	2.673400000
C	-3.622369000	3.758246000	4.564371000
C	-3.275336000	1.483525000	3.801470000
C	-4.615407000	3.054744000	2.453541000
C	-4.371940000	4.030506000	3.428676000
C	-3.068477000	2.496186000	4.739123000
H	-4.780660000	5.032742000	3.278293000
H	-2.455312000	2.294890000	5.618557000
H	-3.455573000	4.538528000	5.309764000

C	2.267467000	-2.885911000	1.892536000
C	0.064044000	-3.445892000	3.485178000
C	1.251015000	-3.702497000	1.367016000
C	2.201274000	-2.352690000	3.199934000
C	1.107177000	-2.684547000	3.996694000
C	0.133546000	-3.928069000	2.186522000
H	1.058377000	-2.313776000	5.022437000
H	-0.683823000	-4.536775000	1.791115000
H	-0.806948000	-3.668710000	4.105818000
C	5.901315000	-0.508878000	-0.385101000
C	7.441060000	1.496650000	-1.532666000
C	6.178286000	0.662066000	0.363220000
C	6.424488000	-0.709597000	-1.673692000
C	7.199376000	0.322870000	-2.222409000
C	6.940650000	1.656503000	-0.245581000
H	7.601401000	0.195481000	-3.231246000
H	7.143023000	2.585272000	0.284847000
H	8.020954000	2.297090000	-1.996362000
C	1.278924000	-4.491299000	0.061244000
H	0.223450000	-4.548771000	-0.251758000
C	1.745537000	-5.926471000	0.352433000
H	2.794781000	-5.931623000	0.689490000
H	1.684058000	-6.543628000	-0.557216000
H	1.138420000	-6.404289000	1.135657000

C	2.055942000	-3.913755000	-1.115736000
H	3.135729000	-4.062644000	-0.994439000
H	1.867426000	-2.835873000	-1.242995000
H	1.754891000	-4.430213000	-2.040150000
C	3.302602000	-1.480179000	3.778982000
H	3.968085000	-1.189330000	2.958638000
C	2.752334000	-0.180666000	4.372354000
H	2.101527000	0.323059000	3.641217000
H	3.580672000	0.496934000	4.633103000
H	2.170460000	-0.359328000	5.290904000
C	4.141775000	-2.262443000	4.795043000
H	3.529122000	-2.591564000	5.649538000
H	4.957584000	-1.635674000	5.186735000
H	4.592175000	-3.157277000	4.338961000
C	6.290254000	-1.953530000	-2.554377000
H	6.314565000	-1.555512000	-3.582219000
C	5.704730000	0.837808000	1.802130000
H	4.634478000	0.574744000	1.828325000
C	6.461926000	-0.093205000	2.761363000
H	6.111385000	0.063612000	3.793275000
H	7.541118000	0.126565000	2.734959000
H	6.326275000	-1.156851000	2.527830000
C	5.826816000	2.272283000	2.315492000
H	5.311462000	2.361242000	3.283306000

H	5.376243000	2.992806000	1.621079000
H	6.879297000	2.555682000	2.477950000
C	5.011472000	-2.782723000	-2.454156000
H	5.030203000	-3.461543000	-1.592166000
H	4.912652000	-3.409266000	-3.353576000
H	4.111696000	-2.159477000	-2.378908000
C	7.529994000	-2.849027000	-2.410728000
H	7.584406000	-3.296541000	-1.406380000
H	8.458809000	-2.283818000	-2.576397000
H	7.494437000	-3.674003000	-3.139081000
C	-3.778293000	-3.707676000	0.848384000
H	-2.979109000	-4.398706000	0.529357000
C	-6.232431000	-0.165998000	-2.051946000
H	-5.596515000	0.609919000	-1.601695000
C	-4.871747000	-4.562145000	1.510473000
H	-5.701065000	-3.929425000	1.864117000
H	-4.466595000	-5.096534000	2.383666000
H	-5.289300000	-5.304883000	0.815539000
C	-3.148428000	-2.780977000	1.883491000
H	-3.893491000	-2.161161000	2.398173000
H	-2.373811000	-2.126068000	1.457645000
H	-2.670210000	-3.394442000	2.659918000
C	-7.592263000	-0.159565000	-1.335115000
H	-8.230073000	-0.971051000	-1.719919000

H	-8.109576000	0.794429000	-1.519709000
H	-7.507621000	-0.276019000	-0.248584000
C	-6.461120000	0.218894000	-3.513751000
H	-6.788321000	1.267747000	-3.574503000
H	-7.254578000	-0.392691000	-3.971999000
H	-5.548871000	0.102218000	-4.109643000
C	-2.620979000	0.123511000	3.993766000
H	-2.535841000	-0.343235000	3.001740000
C	-1.194360000	0.229354000	4.537489000
H	-0.705607000	-0.752257000	4.473489000
H	-1.171959000	0.547466000	5.592257000
H	-0.586990000	0.926567000	3.943285000
C	-3.469742000	-0.789978000	4.885923000
H	-2.989019000	-1.774899000	4.989063000
H	-4.476580000	-0.952377000	4.475160000
H	-3.581027000	-0.357651000	5.893342000
C	-5.327789000	3.527079000	1.190374000
H	-5.855470000	4.446470000	1.492185000
C	-4.274395000	3.931973000	0.151722000
H	-3.614623000	3.089569000	-0.095556000
H	-3.626321000	4.735855000	0.529953000
H	-4.749329000	4.279653000	-0.778130000
C	-6.394633000	2.612675000	0.586867000
H	-7.109949000	2.256562000	1.342428000

H	-5.963025000	1.740617000	0.081872000
H	-6.961531000	3.172594000	-0.172227000
C	-2.496975000	-0.429253000	-2.217256000
C	-2.654000000	-1.139524000	-4.930701000
C	-2.056338000	-1.697943000	-2.659996000
C	-3.031737000	0.464688000	-3.173540000
C	-3.073631000	0.130229000	-4.539958000
C	-2.155806000	-2.064709000	-4.015411000
H	-2.691762000	-1.408814000	-5.991530000
C	-0.922864000	2.061934000	-0.645951000
C	0.178164000	4.604219000	-1.207758000
C	-0.245896000	2.316023000	-1.874109000
C	-0.861085000	3.079829000	0.336193000
C	-0.345720000	4.359390000	0.055691000
C	0.271082000	3.594437000	-2.162884000
H	0.575616000	5.597427000	-1.441077000
C	-3.495841000	1.844521000	-2.776398000
H	-3.676377000	1.914752000	-1.697656000
H	-4.430063000	2.129645000	-3.283429000
H	-2.743963000	2.614767000	-3.017638000
C	-3.491392000	1.137343000	-5.582483000
H	-4.525960000	1.491286000	-5.442463000
H	-3.418152000	0.714604000	-6.594610000
H	-2.849120000	2.032601000	-5.545338000

C	-1.384952000	-2.674732000	-1.724409000
H	-1.605196000	-2.455945000	-0.672635000
H	-0.289373000	-2.607924000	-1.830160000
H	-1.681698000	-3.714807000	-1.925865000
C	-1.692063000	-3.415471000	-4.498215000
H	-2.269922000	-4.231051000	-4.031596000
H	-0.633171000	-3.594617000	-4.251442000
H	-1.802909000	-3.507881000	-5.588130000
C	0.023535000	1.245959000	-2.903595000
H	-0.108845000	0.236618000	-2.504550000
H	1.066573000	1.319890000	-3.237511000
H	-0.610988000	1.344756000	-3.799217000
C	0.949100000	3.882638000	-3.477508000
H	0.325777000	3.589024000	-4.336951000
H	1.898149000	3.329383000	-3.555850000
H	1.183938000	4.952208000	-3.574025000
C	-0.330487000	5.438153000	1.108517000
H	-1.336614000	5.632330000	1.516052000
H	0.056737000	6.383560000	0.702090000
H	0.303385000	5.160203000	1.968033000
C	-1.264060000	2.810466000	1.755576000
H	-1.627176000	1.784993000	1.874636000
H	-2.029921000	3.504374000	2.128726000
H	-0.393973000	2.910642000	2.427424000

C	3.191729000	1.607635000	-0.758507000
C	4.332179000	4.003649000	-1.680988000
C	3.720736000	1.704848000	-2.066694000
C	3.202232000	2.763908000	0.055907000
C	3.751598000	3.971880000	-0.414502000
C	4.327044000	2.891069000	-2.518996000
H	4.778837000	4.937359000	-2.039011000
C	2.577945000	2.769906000	1.429689000
H	3.206735000	3.275764000	2.177504000
H	2.363324000	1.753175000	1.786655000
H	1.615235000	3.302330000	1.400952000
C	3.694178000	5.226857000	0.418197000
H	2.653605000	5.477749000	0.680243000
H	4.123073000	6.083573000	-0.121233000
H	4.242872000	5.121379000	1.369434000
C	3.622953000	0.558551000	-3.044271000
H	3.115074000	0.867718000	-3.973019000
H	3.041038000	-0.280248000	-2.630266000
H	4.611987000	0.179025000	-3.341347000
C	4.927331000	2.979423000	-3.897680000
H	5.685009000	2.195323000	-4.058623000
H	5.408396000	3.954350000	-4.062089000
H	4.164264000	2.847705000	-4.683559000

bis(trimethylsilyl)acetylene, ωB97X-D/Def2-SVP

Geometry of the ground state

Energy = –894.207681105E_h

Si	-2.463725000	0.000093000	0.000049000
Si	2.463674000	0.000104000	-0.000012000
C	0.611721000	0.001207000	0.000614000
C	-0.611772000	0.000836000	0.000426000
C	3.057210000	1.671180000	-0.619306000
H	2.694081000	1.864303000	-1.640432000
H	4.158174000	1.711724000	-0.634013000
H	2.693699000	2.483039000	0.029241000
C	-3.055606000	-1.671380000	0.619900000
H	-4.156521000	-1.712677000	0.635918000
H	-2.691178000	-1.864189000	1.640620000
H	-2.692215000	-2.483017000	-0.028991000
C	3.056475000	-0.299758000	1.757033000
H	2.692898000	0.487785000	2.434862000
H	4.157407000	-0.307324000	1.800241000
H	2.692651000	-1.267438000	2.135318000
C	-3.055744000	0.298417000	-1.757488000
H	-4.156684000	0.304006000	-1.801089000
H	-2.690605000	-0.488785000	-2.434867000
H	-2.693493000	1.266601000	-2.135988000
C	3.055088000	-1.372328000	-1.137996000

H	2.690991000	-2.352789000	-0.794495000
H	4.156016000	-1.406578000	-1.166049000
H	2.691425000	-1.216233000	-2.165247000
C	-3.057312000	1.372362000	1.137108000
H	-2.693336000	2.352996000	0.793974000
H	-2.694674000	1.216506000	2.164763000
H	-4.158281000	1.406087000	1.163969000

IDip, ωB97X-D/Def2-SVP

Geometry of the ground state

Energy = –1158.88044809E$_h$

N	-1.057472000	-0.047372000	0.580148000
N	1.057556000	0.048195000	0.580188000
C	0.000061000	0.000294000	-0.278396000
C	0.675711000	0.034581000	1.915683000
H	1.394687000	0.070943000	2.729231000
C	-0.675694000	-0.033402000	1.915659000
H	-1.394709000	-0.069575000	2.729181000
C	2.425115000	0.105581000	0.161833000
C	-1.870899000	3.000356000	-0.758941000
H	-2.630422000	3.225053000	-1.524777000
H	-1.150747000	2.290241000	-1.190570000
H	-1.337993000	3.934618000	-0.520536000
C	2.966088000	1.342723000	-0.220776000

C	3.179495000	-1.082193000	0.164105000
C	2.115945000	2.601302000	-0.282954000
H	1.197225000	2.413316000	0.292242000
C	-2.424995000	-0.105402000	0.161776000
C	-2.114629000	-2.600981000	-0.283373000
H	-1.195346000	-2.412287000	0.290684000
C	4.309242000	1.370948000	-0.610671000
H	4.759554000	2.315273000	-0.923919000
C	-2.965331000	-1.342789000	-0.220928000
C	-3.179998000	1.081981000	0.164128000
C	4.518001000	-1.001324000	-0.226309000
H	5.134096000	-1.901863000	-0.239624000
C	-2.530404000	2.417059000	0.498154000
H	-1.726910000	2.223271000	1.225209000
C	5.078765000	0.214037000	-0.608965000
H	6.126799000	0.256731000	-0.913762000
C	1.695124000	2.867956000	-1.733186000
H	1.043704000	3.753470000	-1.794219000
H	1.146891000	2.003857000	-2.136403000
H	2.576491000	3.047831000	-2.369865000
C	-3.485662000	3.422089000	1.142527000
H	-2.926750000	4.307419000	1.481281000
H	-4.003230000	2.991523000	2.013126000
H	-4.250033000	3.777369000	0.433694000

C	-4.518510000	1.000424000	-0.226107000
H	-5.135069000	1.900649000	-0.239314000
C	2.529308000	-2.416914000	0.498427000
H	1.726083000	-2.222611000	1.225648000
C	-5.078678000	-0.215215000	-0.608760000
H	-6.126728000	-0.258457000	-0.913424000
C	2.802760000	3.817557000	0.342665000
H	2.109123000	4.671941000	0.367797000
H	3.686606000	4.135141000	-0.232599000
H	3.127177000	3.610854000	1.373721000
C	1.869195000	-3.000111000	-0.758392000
H	1.149127000	-2.289793000	-1.189833000
H	1.336055000	-3.934167000	-0.519695000
H	2.628376000	-3.225151000	-1.524464000
C	-4.308512000	-1.371694000	-0.610688000
H	-4.758329000	-2.316233000	-0.924018000
C	3.484213000	-3.422315000	1.142749000
H	4.002217000	-2.991856000	2.013142000
H	4.248225000	-3.778119000	0.433794000
H	2.924927000	-4.307294000	1.481804000
C	-1.695258000	-2.868179000	-1.733919000
H	-2.577204000	-3.048674000	-2.369618000
H	-1.043578000	-3.753480000	-1.795226000
H	-1.147746000	-2.004072000	-2.138098000

C	-2.800241000	-3.817185000	0.343663000
H	-3.123403000	-3.610095000	1.375035000
H	-2.106291000	-4.671332000	0.368317000
H	-3.684732000	-4.135306000	-0.230312000

References

1 Hintermann, L. Expedient Syntheses of the *N*-heterocyclic Carbene Precursor Imidazolium Salts IPr·HCl, IMes·HCl and IXy·HCl. *Beilstein J. Org. Chem.* **2007**, *3*, 22, DOI: 10.1186/1860-5397-3-22.

2 Légaré, M.-A.; Bélanger-Chabot, G.; Dewhurst, R. D.; Welz, E.; Krummenacher, I.; Engels, B.; Braunschweig, H. Nitrogen Fixation and Reduction at Boron. *Science* **2018**, *359*, 896–900.

3 Dhara, D.; Endres, L.; Krummenacher, I.; Arrowsmith, M; Dewhurst, R. D.; Engels, B.; Bertermann, R.; Finze, M.; Demeshko, S.; Meyer, F.; Fantuzzi, F.; Braunschweig, H. Synthesis and Reactivity of a Crystalline Neutral Diradical Dialumene. *Angew. Chem. Int. Ed.* **2024**, *63*, e202401052.

4 Harris, R. K.; Becker, E. D.; Cabral de Menezes, S. M.; Goodfellow, R.; Granger, P. NMR Nomenclature. Nuclear Spin Properties and Conventions for Chemical Shifts. *Pure Appl. Chem.* **2001**, *73*, 1795–1818.

5 Kurumada, S.; Takamori, S.; Yamashita, M.; An Alkyl-Substituted Aluminium Anion with Strong Basicity and Nucleophilicity. *Nat. Chem.* **2020**, *12*, 36–39.

6 Sheldrick, G. SHELXT - Integrated Space-Group and Crystal-Structure Determination. *Acta. Cryst.* **2015**, *A71*, 3–8.

7 Sheldrick, G. A Short History of SHELX. *Acta Cryst.* **2008**, *A64*,112–122.

8 Spek, A. L. PLATON SQUEEZE: A Tool for the Calculation of the Disordered Solvent Contribution to the Calculated Structure Factors. *Acta Cryst.* **2015**, *C71*, 9–18.

9 Frisch, M. J.; Trucks, G. W.; Schlegel, H. B.; Scuseria, G. E.; Robb, M. A.; Cheeseman, J. R.; Scalmani, G.; Barone, V.; Mennucci, B.; Petersson, G. A.; Nakatsuji, H.; Caricato, M.; Li, X.; Hratchian, H. P.; Izmaylov, A. F.; Bloino, J.; Zheng, G.; Sonnenberg, J. L.; Hada, M.; Ehara, M.; Toyota, K.; Fukuda, R.; Hasegawa, J.; Ishida, M.; Nakajima, T.; Honda, Y.; Kitao, O.; Nakai, H.; Vreven, T.; Montgomery Jr., J. A.; Peralta, J. E.; Ogliaro, F.; Bearpark, M.; Heyd, J. J.; Brothers, E.; Kudin, K. N.; Staroverov, V. N.; Kobayashi, R.; Normand, J.; Raghavachari, K.; Rendell, A.; Burant, J. C.; Iyengar, S. S.; Tomasi, J.; Cossi, M.; Rega, N.; Millam, J. M.; Klene, M.; Knox, J. E.; Cross, J. B.; Bakken, V.; Adamo, C.; Jaramillo, J.; Gomperts, R.; Stratmann, R. E.; Yazyev, O.; Austin, A. J.; Cammi, R.; Pomelli, C.; Ochterski, J. W.; Martin, R. L.; Morokuma, K.; Zakrzewski, V. G.; Voth, G. A.; Salvador, P.; Dannenberg, J. J.; Dapprich, S.; Daniels, A. D.; Farkas, Ö.; Foresman, J. B.; Ortiz, J. V; Cioslowski, J.; Fox, D. J. *Gaussian 16, Revision C.01*; Gaussian, Inc.: Wallingford CT, 2016.

10 Neese, F.; Wennmohs, F.; Becker, U.; Riplinger, C.; The ORCA quantum chemistry program. *J. Chem. Phys.* **2020**, *152*, 224108.

11 Chai, J.-D.; Head-Gordon, M. Long-range corrected hybrid density functionals with damped atom-atom dispersion corrections. *Phys. Chem. Chem. Phys.* **2008**, *10*, 6615-6620.

12 Weigend, F.; Ahlrichs, R. Balanced basis sets of split valence, triple zeta valence and quadruple zeta valence quality for H to Rn: Design and assessment of accuracy. *Phys. Chem. Chem. Phys.* **2005**, *7*, 3297-3305.

13 GaussView, Version 6.0.16, Dennington, R.; Keith, T. A.; Millam, J. M.; Semichem Inc.; Shawnee Mission, KS (2016).

14 (Cancès, E.; Mennucci, B.; Tomasi, J. A New Integral Equation Formalism for the Polarizable Continuum Model: Theoretical Background and Applications to Isotropic and Anisotropic Dielectrics. *J. Chem. Phys.* **1997**, *107*, 3032–3041.

15 Kelly, C. P.; Cramer, C. J.; Truhlar, D. G. SM6: A Density Functional Theory Continuum Solvation Model for Calculating Aqueous Solvation Free Energies of Neutrals, Ions, and Solute−Water Clusters. *J. Chem. Theory Comput.* **2005**, *1*, 1133–1152.

16 Sparta, M.; Riplinger, C.; Neese, F. Mechanism of Olefin Asymmetric Hydrogenation Catalyzed by Iridium Phosphino-Oxazoline: A Pair Natural Orbital Coupled Cluster Study. *J. Chem. Theory Comput.* **2014**, *10*, 1099–1108.

17 Fantuzzi, F.; Nascimento, M. A. C.; Ginovska, B.; Bullock, R. M.; Raugei, S. Splitting of Multiple Hydrogen Molecules by Bioinspired Diniobium Metal Complexes: A DFT Study. *Dalt. Trans.* **2021**, *50*, 840–849.

18 Knizia, G. Intrinsic Atomic Orbitals: An Unbiased Bridge between Quantum Theory and Chemical Concepts. *J. Chem. Theory Comput.* **2013**, *9*, 4834-4843.

19 Knizia, G.; Klein, J. E. M. N. Electron Flow in Reaction Mechanisms – Revealed from First Principles. *Angew. Chem. Int. Ed.* **2015**, *54*, 5518-5522.

20 Mayer, I. Bond order and valence indices: A personal account. *J. Comput. Chem.* **2007**, *28*, 204-221.

21 Dapprich, S.; Frenking, G. Investigation of Donor-Acceptor Interactions: A Charge Decomposition Analysis Using Fragment Molecular Orbitals. *J. Phys. Chem.* **1995**, *99*, 9352–9362.

22 Thom, A. J. W.; Sundstrom E. J.; Head-Gordon, M. LOBA: a localized orbital bonding analysis to calculate oxidation states, with application to a model water oxidation catalyst. *Phys. Chem. Chem. Phys.* **2009**, *11*, 11297-11304.

23 Lu, T.; Chen, F. Multiwfn: A multifunctional wavefunction analyser. *J. Comput. Chem.* **2012**, *33*, 580-592.
